# Dynamics of chromosome organization in a minimal bacterial cell

**DOI:** 10.3389/fcell.2023.1214962

**Published:** 2023-08-09

**Authors:** Benjamin R. Gilbert, Zane R. Thornburg, Troy A. Brier, Jan A. Stevens, Fabian Grünewald, John E. Stone, Siewert J. Marrink, Zaida Luthey-Schulten

**Affiliations:** ^1^ Department of Chemistry, University of Illinois at Urbana-Champaign, Urbana, IL, United States; ^2^ Molecular Dynamics Group, Groningen Biomolecular Sciences and Biotechnology Institute, University of Groningen, Groningen, Netherlands; ^3^ NVIDIA Corporation, Santa Clara, CA, United States; ^4^ NIH Center for Macromolecular Modeling and Bioinformatics, Beckman Institute, University of Illinois at Urbana-Champaign, Urbana, IL, United States; ^5^ NSF Center for the Physics of Living Cells, Department of Physics, University of Illinois at Urbana-Champaign, Urbana, IL, United States

**Keywords:** whole-cell modeling, chromosome replication, chromosome segregation, brownian dynamics, smc proteins, topoisomerase, Martini model

## Abstract

Computational models of cells cannot be considered complete unless they include the most fundamental process of life, the replication and inheritance of genetic material. By creating a computational framework to model systems of replicating bacterial chromosomes as polymers at 10 bp resolution with Brownian dynamics, we investigate changes in chromosome organization during replication and extend the applicability of an existing whole-cell model (WCM) for a genetically minimal bacterium, JCVI-syn3A, to the entire cell-cycle. To achieve cell-scale chromosome structures that are realistic, we model the chromosome as a self-avoiding homopolymer with bending and torsional stiffnesses that capture the essential mechanical properties of dsDNA in Syn3A. In addition, the conformations of the circular DNA must avoid overlapping with ribosomes identitied in cryo-electron tomograms. While Syn3A lacks the complex regulatory systems known to orchestrate chromosome segregation in other bacteria, its minimized genome retains essential loop-extruding structural maintenance of chromosomes (SMC) protein complexes (SMC-scpAB) and topoisomerases. Through implementing the effects of these proteins in our simulations of replicating chromosomes, we find that they alone are sufficient for simultaneous chromosome segregation across all generations within nested theta structures. This supports previous studies suggesting loop-extrusion serves as a near-universal mechanism for chromosome organization within bacterial and eukaryotic cells. Furthermore, we analyze ribosome diffusion under the influence of the chromosome and calculate *in silico* chromosome contact maps that capture inter-daughter interactions. Finally, we present a methodology to map the polymer model of the chromosome to a Martini coarse-grained representation to prepare molecular dynamics models of entire Syn3A cells, which serves as an ultimate means of validation for cell states predicted by the WCM.

## 1 Introduction

The goal of computational modeling of a single cell is to create whole-cell models (WCMs) that propagate the state of an entire cell through time, where the propagation is governed by the chemical and physical interactions within the cell and between the cell and its environment (Karr et al., 2012; Goldberg et al., 2018; Macklin et al., 2020; Marucci et al., 2020; Luthey-Schulten et al., 2022; Maritan et al., 2022; Thornburg et al., 2022). To model any cell in 3D, configurations and dynamics of the chromosome(s) are critical in defining the spatial heterogeneity of gene expression over the course of a cell-cycle (Llopis et al., 2010). While there are several existing models that can simulate entire bacterial chromosomes (Buenemann and Lenz, 2010; Messelink et al., 2021; Wasim et al., 2021), relatively few are at spatial resolutions less than hundreds to thousands of base pairs (bp) per particle (Hacker et al., 2017; Goodsell et al., 2018; Gilbert et al., 2021). Here, we introduce a computational model to simulate the 3D dynamics of the chromosome of a genetically minimal bacterium, JCVI-syn3A, at 10-bp resolution including replicating chromosome states (Cooper and Helmstetter, 1968; Bremer and Dennis, 2008; Youngren et al., 2014) and loop-extrusion by structural maintenance of chromosomes (SMC) protein complexes (Hirano, 2006; Alipour and Marko, 2012; Ganji et al., 2018; Lioy et al., 2020; Davidson and Peters, 2021; Lee et al., 2021).

JCVI-syn3A is a minimal bacterial cell with a chemically synthesized 543 kbp genome composed of 493 genes (Breuer et al., 2019). The SynX-series of organisms began with JCVI-syn1.0, which was created by transplanting a chemically synthesized *Mycoplasma mycoides* genome into living *Mycoplasma* cells (Gibson et al., 2010). JCVI-syn3.0 was subsequently created by synthetically reducing the 1,079 kbp genome of Syn1.0 until what was considered a genetically minimal bacterium with a 531 kbp genome, stripped of all but the necessary components to continue proliferating, was achieved (Hutchison et al., 2016). Finally, Syn3A was created by re-introducing 19 genes from Syn1.0 back into Syn3.0’s genome. While this produced an arguably less-minimal bacterium, it increased the growth rate (180 min doubling-time in Syn3.0 to 110 min doubling-time in Syn3A) (Breuer et al., 2019) and restored a regular spherical morphology to the cells (Pelletier et al., 2021).

With a genome and physical size approximately one-tenth the size of the model bacterium *Escherichia coli*, Syn3A is ideally suited for whole-cell modeling due to the corresponding reduction in complexity. Syn3A′s initial cell state was defined through experimental charactizations of its biochemical components — genome-wide gene-essentiality and proteomics (Breuer et al., 2019), metabolomics (Haas et al., 2022), lipidomics (Thornburg et al., 2022), and cellular architecture from cryo-electron tomography (cryo-ET) (Gilbert et al., 2021). Systematic investigations of the interactions amongst Syn3A′s biochemical components were undertaken — defining the metabolic map (Breuer et al., 2019), genetic information processes (Thornburg et al., 2019), and reaction kinetics of coupled metabolic/genetic information processes (Thornburg et al., 2022). By combining these with hybrid stochastic-deterministic methods leveraging GPU-accelerated simulation software (Roberts et al., 2012; Hallock et al., 2014; Bianchi et al., 2018), a well-stirred WCM (WS-WCM) and 3D spatially resolved WCM (4D-WCM) that predict time-dependent Syn3A cell states were created (Thornburg et al., 2022).

**FIGURE 1:**
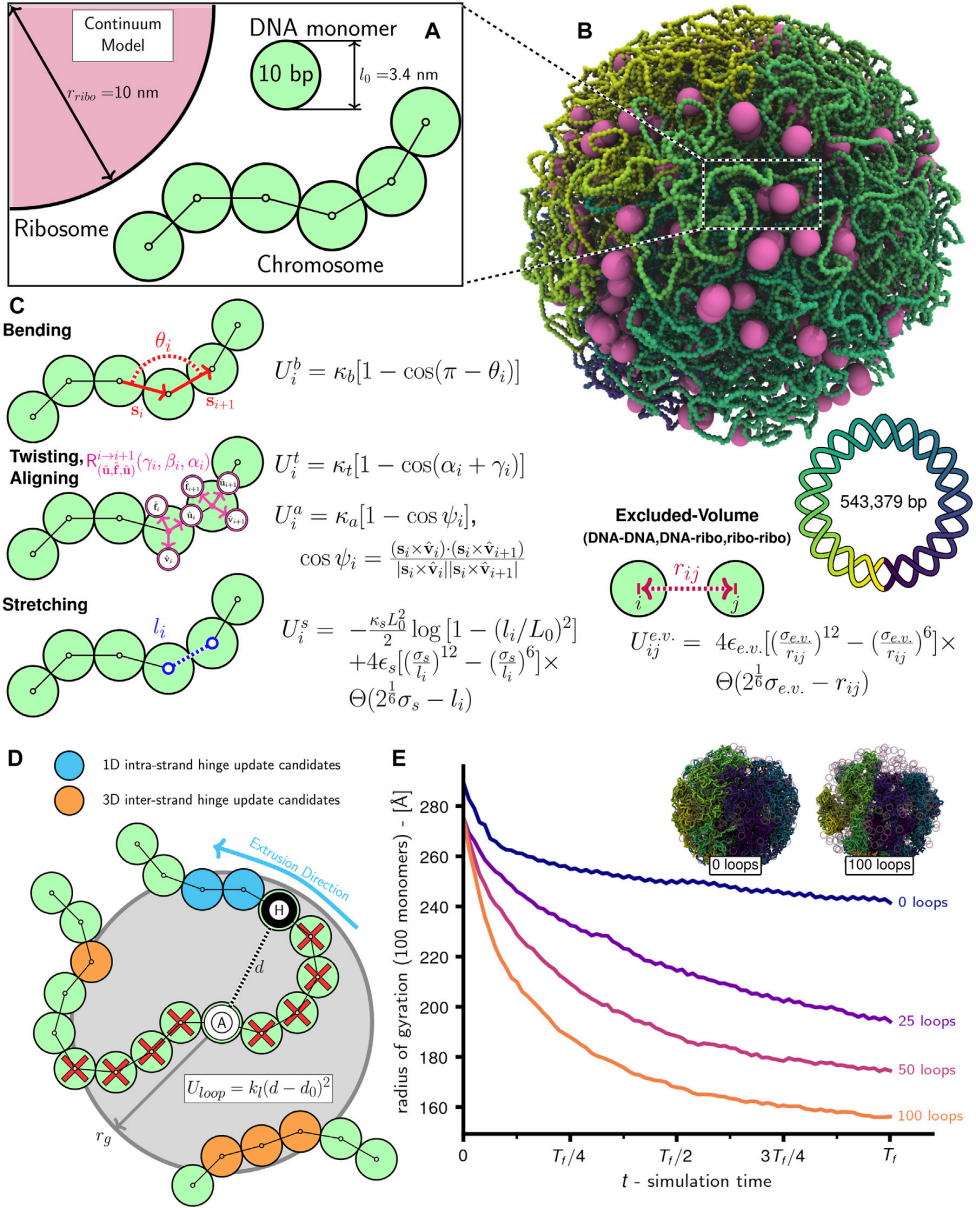
Schematic of chromosome modeling protocol: **(A)** Diagram of system with ribosomes and chromosome comprised of 10 bp DNA monomers. **(B)** Snapshot of system with an unreplicated 54,338 monomer Syn3A chromosome and 500 ribosomes in a 200 nm radius cell. **(C)** Bending $\left(U_{i}^{b}\right)$ , twisting $\left(U_{i}^{t}\right)$ , aligning $\left(U_{i}^{a}\right)$ , and stretching $\left(U_{i}^{s}\right)$ potential energy functions for intramonomer interactions, and potential energy functions for excluded-volume interactions $\left(U_{ij}^{e.v.}\right)$ between DNA monomers and ribosomes. **(D)** DNA loops are created by applying harmonic bonds between pairs of “anchor” (A, white) and “hinge” (H, black) monomers. Loop-extrusion is simulated by periodically updating the hinge monomer from the set of candidates within the grab radius, *r*_*g*_. Monomers with a red cross are excluded from hinge updates due to not satisfying the minimal loop length requirement, *L*_min_. **(E)** Average windowed radius of gyration as a function of time for simulations of a single unreplicated chromosome with varying numbers of loops. Simulations were run for 4.0E + 6 timesteps with parameters given in Section 2.4.4. Inset are snapshots of the simulations with 0 loops and 100 loops at *t* = *T*_*f*_.

**FIGURE 2:**
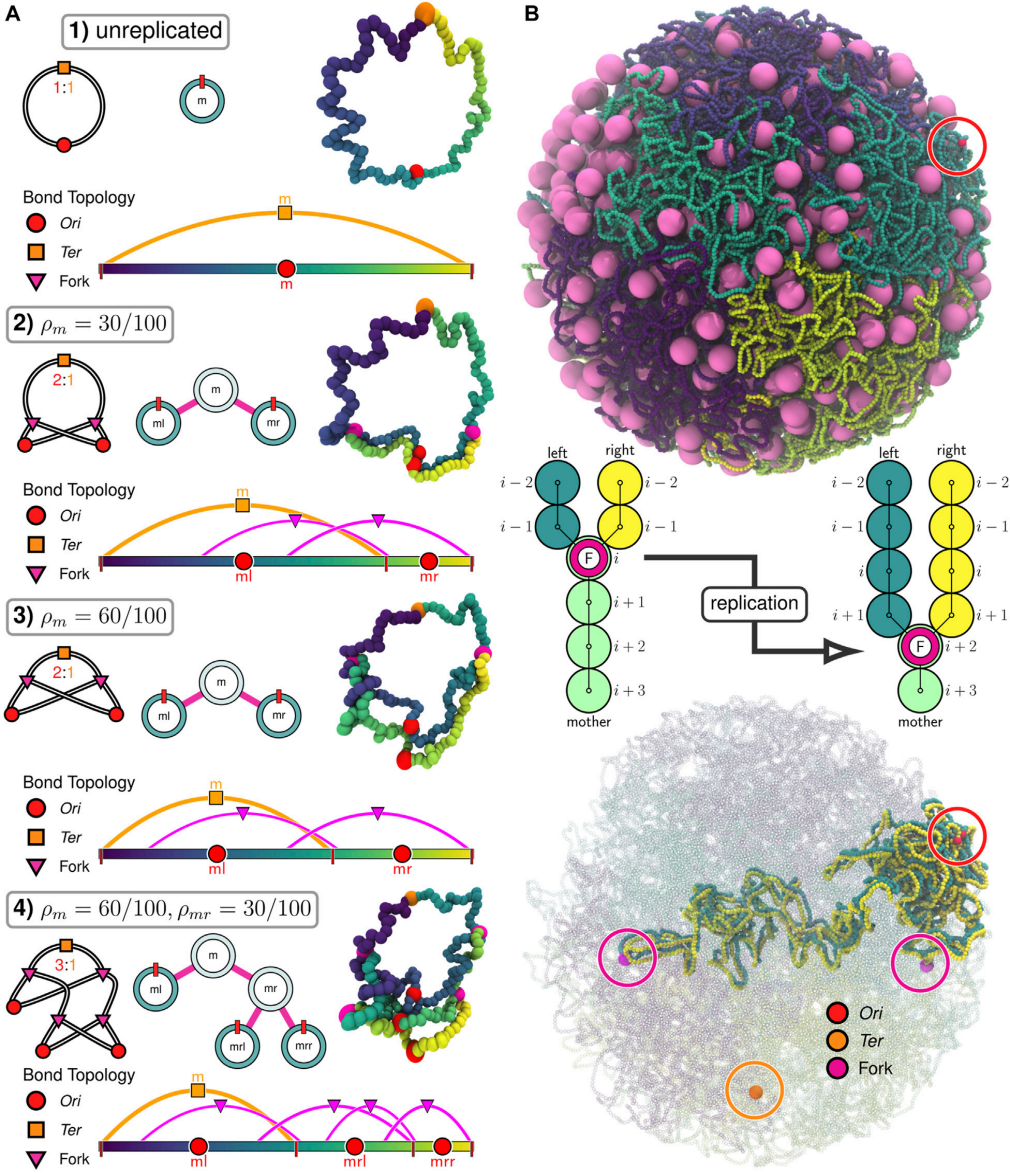
Replication of chromosomes in polymer model: **(A)** Progressive replication $\left(\rho_{i}=\rho_{i}^{cw}+\rho_{i}^{\mathrm{ccw}}\right)$ of 100 monomer circular DNA using binary tree model. For each of the four stages of replication, we show the theta structure in the top-left, the binary tree representation in the top-middle, the physical model in the top-right, and the bond topology of the physical model in the bottom. The bond topology displays all monomers using the colorbar at the bottom. Adjacent monomers in regions of the colorbar partitioned by red lines (chromosome boundaries) are bonded. All other bonds in the system (*Ter*s creating circular chromosomes and forks creating theta structures) are depicted using arcs between the bonded monomers. **(B)** Beginning with an unreplicated Syn3A chromosome (543,379 bp) within a 200 nm radius cell containing 500 ribosomes, 20,000 bp (2,000 monomers) were replicated using the train-track model (see schematic). The *Ori*s, *Ter*, and forks in the replicated system are highlighted with circles.

**FIGURE 3:**
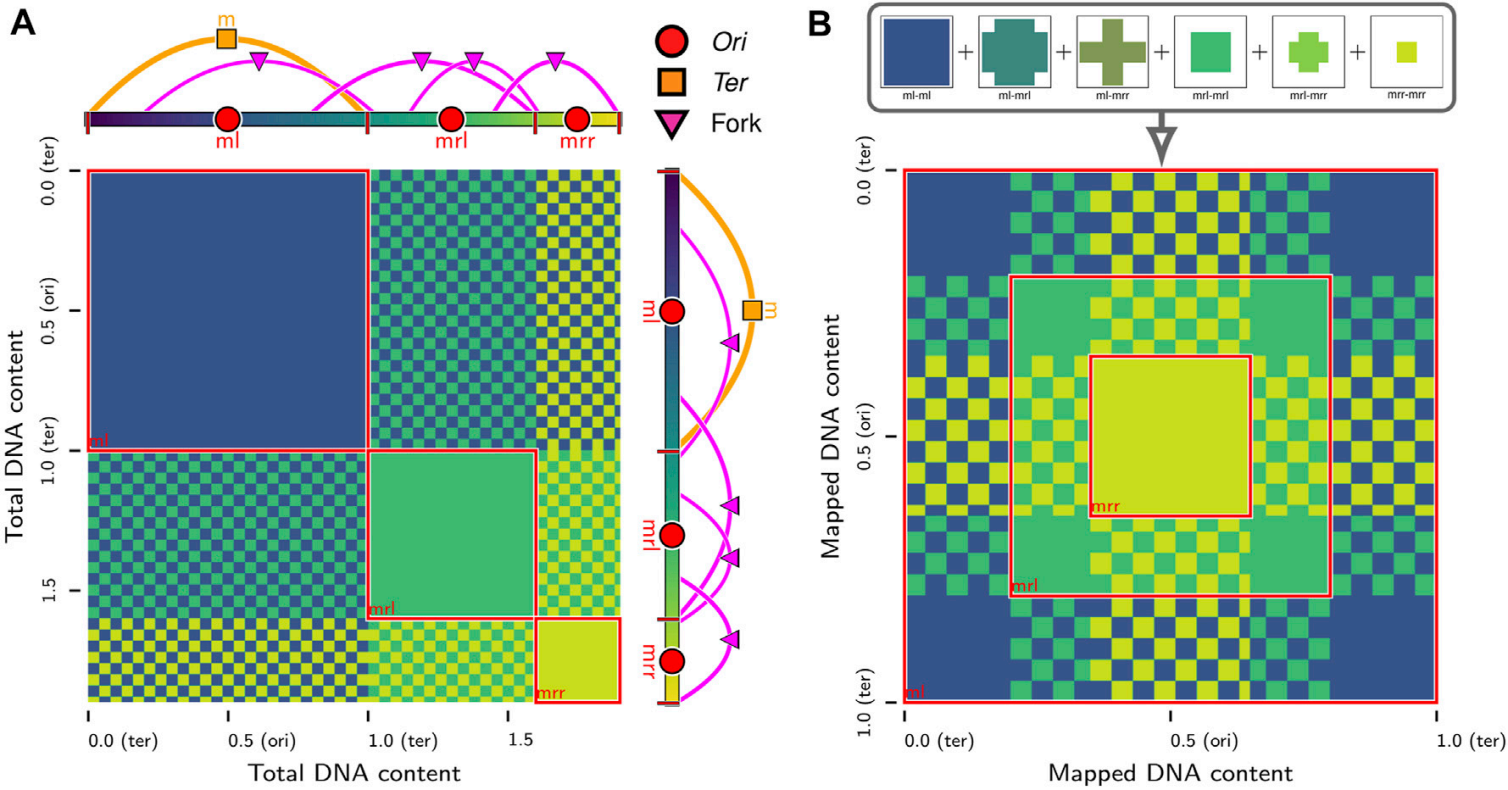
*In silico* contact calculations for replicating chromosomes: **(A)** The true contact map, F , of intra- and inter-daughter interactions in a replicating chromosome system with a nested theta structure. The system presented is replication state 4 in Figure 2A. Solid-colors indicate self-interactions of daughter chromosomes and checkerboard patterns indicate interactions between pairs of daughter chromosomes. **(B)** Mapping of loci in contact map of replicating chromosome system in **(A)** to equivalent loci with identical sequences in unreplicated system. The overlapping patterns are summed to produce the sequence-equivalent contact map, $\tilde{F}$ , that is equivalent to contact maps observed by sequence-based experimental methods.

However, due to the methodology used to model the chromosome (Gilbert et al., 2021), the existing 4D-WCM was limited to the part of the cell-cycle prior to the onset of DNA replication (Thornburg et al., 2022). This study resolves that issue by transitioning from a lattice polymer model to a continuum polymer model (Figure 1A) of the chromosome, while retaining the previous model’s strengths; namely, the ability to fold chromosomes within cellular architectures dictated by cryo-ET and a high spatial resolution (10 bp per monomer) that enables modeling of the hetergeneous diffusion of macromolecular complexes due to excluded-volume interactions with the chromosome. Furthermore, the new method allows for progressive DNA replication of the chromosome to reach nontrivial replication states (Cooper and Helmstetter, 1968; Bremer and Dennis, 2008; Youngren et al., 2014; Khan et al., 2016; Wasim et al., 2021; Pountain et al., 2022) and for the segregation of daughter chromosomes (Goloborodko et al., 2016a; Gogou et al., 2021) under the influence of known essential components (Breuer et al., 2019), SMC-complexes (Ganji et al., 2018; Lee et al., 2021) and topoisomerases (Wang, 1991; McKie et al., 2021; Sutormin et al., 2021; Conin et al., 2022). These nontrivial replication states have *Ori*:*Ter* ratios greater than 2:1 (Figure 2), where *Ori* is the origin of replication and *Ter* is the terminus of replication, and were predicted in Syn3A by WS-WCM simulations and measured by experimental quantitative-PCR (qPCR) (Thornburg et al., 2022). These new capabilities lay the groundwork for the extension of the 4D-WCM to the full cell-cycle. Additionally, by using a binary tree model (Figure 2A) the full spectrum of replication of states for a circular chromosome can be explored and *in silico* chromosome contact maps resolving inter-daughter interactions can be calculated (Figure 3A).

Beyond the information stored in the sequence of the genome, the 3D organization of eukaryotic (Kempfer and Pombo, 2019) and bacterial (Dame et al., 2019; Lioy et al., 2021) genomes plays a role in cellular behavior (Dekker and Mirny, 2016). While imaging techniques such as DNA-FISH (Giorgetti and Heard, 2016) provide insights about targeted interactions, the wide-spread accessibility of next-generation sequencing (Goodwin et al., 2016) catalyzed the proliferation of sequence-based techniques that assess genome-wide interactions such as DNA-protein binding using CHIP-seq (Park, 2009) and DNA-DNA proximity using chromosome conformation capture (3C) (Dekker et al., 2002). Following the creation of 3C, many variations have been developed (Denker and de Laat, 2016; Goel and Hansen, 2020), the most well-known of which is perhaps Hi-C (Lieberman-Aiden et al., 2009). Although researchers have a stunning breadth of experimental data characterizing interactions throughout the genome, computational models (Rosa and Zimmer, 2014; Tiana and Giorgetti, 2019) are required to solve the inverse problem of determining 3D genome organization (Di Pierro et al., 2017; Messelink et al., 2021; Shi and Thirumalai, 2023) and provide mechanistic insights (Sanborn et al., 2015; Fudenberg et al., 2016; Banigan et al., 2020; Fiorillo et al., 2021).

Syn3A is a compelling system for a systematic study of bacterial chromosomes (Birnie and Dekker, 2020), including but not limited to their replication and segregation, and the bacterial cell cycle (Olivi et al., 2021) because the protein functions encoded by its remaining essential genes hypothetically represent the minimal ingredients necessary for successful proliferation of bacterial cells. Based on what is known of chromosome organizing elements, key among these minimal ingredients should be at least one creating DNA loops (DNA regions distant in sequence but constrained in close spatial proximity) (Davidson and Peters, 2021) and one resolving DNA knots and catenanes (McKie et al., 2021). Syn3A′s genome encodes the prokaryotic condensin complex, SMC-scpAB (Table 1), whose *Saccharomyces cerevisiae* homolog extrudes DNA loops at rates of hundreds of bp per second (Ryu et al., 2021), along with two type-II topoisomerases that allow strand-passage of dsDNA (Liu et al., 1980), DNA-gyrase (gyrase) and topoisomerase-IV (topo-IV) (Table 1) — all of these genes were found to be essential by transposon mutagenesis (Breuer et al., 2019). We compare Syn3A′s proteomics counts of SMC-scpAB and type-II topoisomerases with respect to the model bacteria *E. coli* and *B. subtilis* on the basis of their counts relative to the total DNA content of the genome, as the DNA is what these proteins manipulate. After accounting for the 4:2:4 stoichiometry (Lee et al., 2021) of *E. coli*′s SMC complex, MukBEF, we find that the densities of SMC core proteins per bp of genome are ranked in decreasing order as 1) *E. coli*, 2) Syn3A, 3) *B. subtilis* (Table 1). However, the difference between *E. coli* and *B. subtilis* is only one order-of-magnitude and we can conjecture that this might be due to Syn3A and *E. coli* compensating for their lack of a parAB*S* system (Livny et al., 2007; Badrinarayanan et al., 2015) that preferentially loads SMC complexes onto the chromosome (Marbouty et al., 2015; Tran et al., 2017). We find similar trends among the densities of the two type-II topoisomerases (Table 1). Given the comparable densities of these chromosome-manipulating proteins between Gram-positive (Syn3A and *B. subtilis*) and -negative (*E. coli*) bacteria, we feel that Syn3A is a suitable system in which to study the dynamics of bacterial chromosome organization.

**TABLE 1:** Comparative proteomics of proteins in Syn3A that are known to interact with bacterial chromosomes (SMC-scpAB, DNA-gyrase, topoisomerase-IV, and HU). Values were taken from Supplementary Table S1 of (Thornburg et al., 2022).

Protein (stoichiometry)	Locus	# Syn3A	#/Genome size [#/bp]		
			Syn3A	*E. coli* ^a^	*B. subtilis* ^b^
SMC (2)	0415	202	3.72E-4	2.18E-3^c^	1.07E-4
ScpA (1)	0327	1 (10)^d^	1.84E-5	-	-
ScpB (2)	0328	31	5.71E-5	-	1.88E-5
gyrase-A (2)	0007	298	5.48E-4	1.86E-3	3.05E-4
gyrase-B (2)	0006	244	4.49E-4	1.32E-3	1.67E-4
topoIV-A (2)	0453	156	2.87E-4	2.77E-4	4.83E-5
topoIV-B (2)	0452	157	2.89E-4	1.38E-4	4.71E-5
HU (2)^e^	0350	28	5.15E-5	2.69E-3	2.01E-3

^a^ 4.6 Mbp genome.

^b^ 4.4 Mbp genome.

^c^ SMC complex in *E. coli* is MukBEF, with stoichiometry of 4:2:4.

^d^ Proteins with counts less than 10 were assumed to be a minimum of 10 in whole-cell simulations.

^e^ Greatest %-identity with *α*-subunit (HU*α*), HU, forms homo- (*αα* or *ββ*) and heterodimers (*αβ*) in *E. coli*.

As was noted in a previous study (Gilbert et al., 2021), unlike many bacteria Syn3A codes for a single nucleoid-associated protein (NAP) (Dame, 2005; Lioy et al., 2018; Verma et al., 2019; Lioy et al., 2021), HU (JCVISYN3A_0350), which is known to have two binding modes: 1) low-affinity binding to linear DNA and 2) high-affinity binding to structurally deformed DNA (Kamashev, 2000; Verma et al., 2023). One result of HU binding is the stabilization of supercoiling (Le et al., 2013; Lioy et al., 2018; Strzałka et al., 2022). Curiously, while the HU gene was found to be essential by transposon mutagenesis, the proteomics count is so vastly lower than that of *E. coli*, *B. subtilis*, and related-organism *Mesoplasma florum* (Gilbert et al., 2021) that its genome-wide influence (Pelletier et al., 2012) is likely to be negligible. Furthermore, chromosome contact maps from 3C-seq libraries of Syn3A do not exhibit chromosome interaction domains (CIDs) (Gilbert et al., 2021), which are known to be a result of persistent supercoiling (Le et al., 2013; Trussart et al., 2017; Lioy et al., 2018). Given these considerations, we hypothesize that HU’s lingering essentiality in Syn3A is a reflection of it only acting through an interaction specific to the high-affinity binding mode. In *E. coli*, HU is known to interact with replication initiator protein DnaA (Chodavarapu et al., 2007), HU*α*/DNA stoichiometry has been shown to increase for faster-growing *E. coli* cells (Abebe et al., 2017), and experimental evidence suggests a mechanism of HU promoting duplex unwinding at the *oriC* replication origin (Yoshida et al., 2023). Additionally, HU is essential for replication initiation in Gram-positive *B. subtilis* (Karaboja and Wang, 2022; Schramm and Murray, 2022), whose replication origin is similarly a DnaA-based *oriC*. Based on these results in other bacteria and HU’s enhanced binding to dsDNA repair and recombination intermediates (Kamashev, 2000), we believe a small number of HU was retained to fulfill an essential role during replication initiation using a DnaA-based *oriC* in Syn3A (Thornburg et al., 2019), but do not expect it to influence chromosome-scale organization with its reduced proteomics count, and therefore exclude HU from our model of the chromosome.

Given the absence of NAPs structuring Syn3A′s chromosome into bacterial chromatin (Dame and Tark-Dame, 2016), we chose to model the chromosome of essentially naked dsDNA as a twistable and elastic worm-like chain (Klenin et al., 1998; Brackley et al., 2014; Maffeo and Aksimentiev, 2020). The polymer is comprised of 3.4 nm diameter spherical monomers containing 10 bp of chromosomal DNA (Figure 1A), 54,338 such monomers bonded in a circle are used to create Syn3A′s 543,379 bp circular chromosome (Figure 1B). Adjacent monomers interact through stretching, bending, and twisting potentials (Figure 1C) that reproduce the tensile, bending, and torsional stiffness of dsDNA (Cocco et al., 2002; Brackley et al., 2014), and are parameterized by linear (45 nm) (Manning, 2006; Geggier et al., 2010; Mantelli et al., 2011) and twist (85 nm) (Mosconi et al., 2009) persistence lengths. The monomers are subject to non-bonded interactions that prevent strand-crossings and cause them to avoid ribosomes modeled as 20 nm diameter spherical particles (Figure 1C). We chose to neglect electrostatics and hydrodynamic interactions in this current model. The complete system of chromosomes and ribosomes is simulated using a Brownian dynamics (Snook, 2007) integrator for aspherical particles in LAMMPS (Thompson et al., 2022). To explore the influence of loop-extruding SMC complexes and strand-crossing type-II topoisomerase in this framework, we have developed algorithms to selectively introduce and remove additional terms in the energy function that emulate their effects.

While the computational methodologies described in this paper are tailored to reaching the longer-timescales necessary for WCMs that include fundamental processes of bacterial life such as chromosome replication and segregation, returning to the near-atomic scale provides the ultimate means of validation and reveals additional insights. Researchers have previously completed molecular dynamics (MD) simulations of representative volumes of bacterial cytoplasm (Yu et al., 2016; Rickard et al., 2019; Heo et al., 2022), but only recently has it become possible to prepare a MD simulation of an entire bacterium (Stevens et al., 2023). We will describe how our polymer model for the chromosome can be directly mapped to a coarse-grained Martini model (Marrink et al., 2022) of dsDNA that is ready to be simulated using Gromacs-2023 (Páll et al., 2020; Abraham et al., 2023).

## 2 Materials and methods

### 2.1 Twistable polymer model

The chromosome is modeled under the assumption that due to the low density of NAPs in Syn3A, the vast majority of the chromosome is essentially naked dsDNA in a good solvent (Breuer et al., 2019; Szatmári et al., 2020; Thornburg et al., 2022). The naked dsDNA is represented as a twistable and elastic worm-like chain of spherical monomers, each of which contain 10 bp of DNA and have a radius (*r*_DNA_) of 1.7 nm. We model the 10 bp monomers as spheres rather than 3.4 nm cylindrical segments with diameters equal to that of a dsDNA helix (2 nm) because using isotropic pair potentials for spherical particles is less computationally intensive. We consider the spherical monomer approximation acceptable for our chromosome-scale model because relative to a cylindrical segment the excluded volume is overestimated by less than a factor of two and the translational damping (Section 2.2) is overestimated by only 15% (Supplementary Analyses). Monomers interact through the energy function from Brackley et al. (Brackley et al., 2014) — the monomers are bonded using finitely extensible nonlinear elastic (FENE) potentials (*l*_*i*_ in Figure 1C), the bending stiffness of dsDNA is implemented using a cosine potential whose argument is the angle (*θ*_*i*_ in Figure 1C) between (*i* − 1)-, *i*-, and (*i* + 1)-th monomers, and the torsional stiffness of dsDNA is implemented using a cosine potential whose argument is the sum of Euler angles parameterizing the rotation matrix describing the transformation between the local coordinate systems, $\left({\hat{u}}_{i},{\hat{f}}_{i},{\hat{v}}_{i}\right)$ , of *i*- and (*i* + 1)-th monomers (*α*_*i*_ and *γ*_*i*_ in Figure 1C). The linear and torsional stiffness parameters, *κ*_*b*_ and *κ*_*t*_, are determined based on the assumed linear persistence length, *l*_*p*_, of 45 nm (Manning, 2006; Geggier et al., 2010; Mantelli et al., 2011) and twist persistence length, *l*_*t*_, of 85 nm (Mosconi et al., 2009), respectively. The alignment term in the potential serves to align the ${\hat{u}}_{i}$ basis vector of the *i*-th monomer’s local coordinate system with the displacement vector between the *i*- and (*i* + 1)-th monomers, ***s***_*i*_. While the monomer orientations and torsional interactions play a limited role in the current simulations due the assumption of a relaxed supercoiling state, we elected to include them for a few reasons. First, the train-track model of replication (Section 2.6) uses the monomer orientations to specify the coordinates of the daughter chromosomes (Figure 2B). Second, in the future we intend to use the model to investigate chromosome organization due to DNA-binding HU (Lioy et al., 2018) when its expression is restored and to mechanochemically couple transcriptional activity in the 4D-WCM to the torsional state of the chromosome (Liu and Wang, 1987; Chong et al., 2014; Dorman, 2019; Kim et al., 2019; Chatterjee et al., 2021; Guo et al., 2021; Geng et al., 2022).

The chromosome as a whole is modeled as a homopolymer and all monomers, including those representing the *Ori*s, *Ter*s, and forks, have an identical radius of *r*_DNA_. The ribosomes are modeled as spheres with a radius (*r*_ribo_) of 10.0 nm. Not pictured in Figure 1 are boundary particles with a radius (*r*_bdry_) of 5*r*_DNA_ that create the closed membrane shape. All non-bonded particles interact through purely repulsive Weeks-Chandler-Andersen (WCA) pair potentials (Figure 1C), which serve to prevent dsDNA strand-crossings (Supplementary Video SV1), create the excluded-volume interactions between the chromosome and ribosomes, and confine all DNA monomers and ribosomes within the surface comprised of boundary particles.

The total potential energy function for the chromosome/ribosome system is

$$\begin{array}{cc} U= & \overset{N_{\text{DNA}}}{\underset{i=1}{\sum }}\left[U_{i}^{b}+U_{i}^{t}+U_{i}^{a}+U_{i}^{s}\right]+\\ & +\overset{N_{\text{DNA}}-1}{\underset{i=1}{\sum }}\overset{N_{\text{DNA}}}{\underset{j=i+1}{\sum }}U_{ij}^{\text{DNA}-\text{DNA}}+\overset{N_{\text{DNA}}}{\underset{i=1}{\sum }}\overset{N_{\text{ribo}}}{\underset{j}{\sum }}U_{ij}^{\text{DNA}-\text{ribo}}\\ & +\overset{N_{\text{ribo}}-1}{\underset{i=1}{\sum }}\overset{N_{\text{ribo}}}{\underset{j=i+1}{\sum }}U_{ij}^{\text{ribo}-\text{ribo}}\\ & +\overset{N_{\text{bdry}}}{\underset{i=1}{\sum }}\overset{N_{\text{DNA}}}{\underset{j}{\sum }}U_{ij}^{\text{bdry}-\text{DNA}}+\overset{N_{\text{bdry}}}{\underset{i=1}{\sum }}\overset{N_{\text{ribo}}}{\underset{j}{\sum }}U_{ij}^{\text{bdry}-\text{ribo}}\text{,} \end{array}$$ (2.1)

where the details of the energy functions may be found in Figure 1A. Soft pair potentials of the form

$$U_{ij}^{\text{soft/topo}}=\epsilon_{\text{soft/topo}}\left[1+\cos\left(\frac{\pi r_{ij}}{\sigma_{ij}}\right)\right],\;\text{where}\;r_{ij}<\sigma_{ij}$$ (2.2)

are used to reduce overlaps during energy minimizations (replacing $U_{ij}^{\text{DNA}-\text{DNA}}$ and $U_{ij}^{\text{DNA}-\text{ribo}}$ ) and permit strand-crossings of DNA (Supplementary Video SV1) under the assumed action of topoisomerases (replacing $U_{ij}^{\text{DNA}-\text{DNA}}$ ). Additionally, the FENE bonds between monomers are replaced with harmonic bonds of the form

$$U_{i}^{s}=k_{\text{min}}{\left(l_{i}-l_{0}\right)}^{2}$$ (2.3)

during the initial energy minimizations to prevent over-stretching. Excluding the SMC looping interactions, which are described in greater detail in Section 2.3, all energetic parameters for the potential energy function are listed in Table 2.

**TABLE 2:** Potential energy parameters for the chromosome and ribosome system. All simulation units are using “units real” in LAMMPS (Thompson et al., 2022).

Parameter	Symbol	Simulation units	
		Quantity	Unit
DNA monomer radius	*r*_DNA_	1.7E+1	Å
ribosome radius	*r*_ribo_	1.0E+2	Å
boundary particle radius	*r*_bdry_	2.5*r*_DNA_	Å
eq. monomer spacing	*l*_0_	2*r*_DNA_	Å
linear persistence length	*l*_*p*_	4.5E+2	Å
twist persistence length	*l*_*t*_	8.5E+2	Å
bending energy	*κ*_*b*_/*k*_*B*_ *T*	*l*_*p*_/(2*r*_DNA_)	n.d.
twisting energy	*κ*_*t*_/*k*_*B*_ *T*	*l*_*t*_/(2 × (2*r*_DNA_))	n.d.
aligning energy	*κ*_*a*_	2*κ*_*t*_	Kcal/mol
FENE rep. energy	*ϵ*_*s*_/*k*_*B*_ *T*	1.0	n.d.
FENE rep. length	*σ*_*s*_	2*r*_DNA_	Å
FENE att. energy	$\kappa_{s}\sigma_{s}^{2}/k_{B}T$	1.0E+2	n.d.
FENE finite-length	*L*_0_	1.5*σ*_*s*_	Å
DNA-DNA WCA energy	*ϵ*_DNA-DNA_/*k*_*B*_ *T*	1.0	n.d.
DNA-DNA WCA length	*σ*_DNA-DNA_	2*r*_DNA_	Å
DNA-ribo WCA energy	*ϵ*_DNA-ribo_/*k*_*B*_ *T*	1.0	n.d.
DNA-ribo WCA length	*σ*_DNA-ribo_	*r*_DNA_ + *r*_ribo_	Å
ribo-ribo WCA energy	*ϵ*_ribo-ribo_/*k*_*B*_ *T*	1.0	n.d.
ribo-ribo WCA length	*σ*_ribo-ribo_	2*r*_ribo_	Å
bdry-DNA WCA energy	*ϵ*_bdry-DNA_/*k*_*B*_ *T*	1.0	n.d.
bdry-DNA WCA length	*σ*_bdry-DNA_	*r*_bdry_ + *r*_DNA_	Å
bdry-ribo WCA energy	*ϵ*_bdry-ribo_/*k*_*B*_ *T*	1.0	n.d.
bdry-ribo WCA length	*σ*_bdry-ribo_	*r*_bdry_ + *r*_ribo_	Å
soft pairs	*ϵ*_soft_/*k*_*B*_ *T*	1.0	n.d.
topoisomerase pairs	*ϵ*_topo_/*k*_*B*_ *T*	1.0E-1	n.d.
minimization bond energy	$k_{\text{min}}l_{0}^{2}/k_{B}T$	1.0E+3	n.d.

### 2.2 Brownian dynamics

The time-integration was carried out using an OpenMP-accelerated version of the Brownian dynamics integrator for aspherical particles (Delong et al., 2015; Ilie et al., 2015) in LAMMPS (Thompson et al., 2022). The Brownian equation of motion

$$\frac{dx_{i}}{dt}=\frac{F_{\text{system}}+F_{\text{random}}}{\gamma_{i}}$$ (2.4)

approximates the overdamped limit of the Langevin equation

$$\frac{m_{i}}{\gamma_{i}}\frac{d^{2}x_{i}}{dt^{2}}=-\frac{dx_{i}}{dt}+\frac{F_{\text{system}}+F_{\text{random}}}{\gamma_{i}}\text{,}$$ (2.5)

and is only an accurate approximation if the inertial forces are insignificant compared to the viscous forces (Snook, 2007). The mass of the 10 bp monomers is sequence-independent and was calculated as the molar mass of an average 10 bp sequence from Syn3A′s genome (Breuer et al., 2019). We model only complete 70S ribosomes with an assumed mass of 2,700 KDa (Yamamoto et al., 2006). Both ribosomes and DNA monomers are assumed to behave as spherical particles undergoing normal Brownian motion in a Newtonian fluid. In the case of the ribosomes, their characteristic size is 20 nm when we do not include polysomes (multiple ribosomes translating a single mRNA) (Xue et al., 2022), and their motion should be decoupled from metabolic activity due to falling below a 30 nm size threshold (Parry et al., 2014). Although the chromosome is a cytoplasmic component with size well in excess of this threshold, we model the DNA monomers under the same simplifying assumption of normal Brownian motion. In reality, bacterial chromosome dynamics are a result of ATP-dependent motion (Weber et al., 2012), and part of this motion originates from loop-extrusion by SMC (Hirano, 2006), which is addressed by another part of our computational model (Section 2.3). The damping coefficients for the translational and rotational motion of DNA monomers and ribosomes are listed in Table 3. Translational damping constants, $\gamma_{i}^{\text{T}}$ , were calculated using the Stokes-Einstein equation for spherical particles (Snook, 2007)

$$\gamma_{i}^{\text{T}}=6\pi \eta r_{i}$$ (2.6)

with the dynamic viscosity used previously in the 4D-WCM (Thornburg et al., 2022). Rotational damping constants, $\gamma_{i}^{\text{R}}$ , were calculated assuming no-slip boundary conditions between the spherical solute particles and surrounding solvent

$$\gamma_{i}^{\text{R}}=\frac{\gamma_{i}^{\text{T}}r_{i}^{2}}{3}\text{.}$$ (2.7)

the timestep, Δ*t* = 0.1 ns, was selected such that it satisfies the conditions of the overdamped limit of the Langevin equation, $\Delta t\gg m_{i}/\gamma_{i}^{\text{T}}$ and $\Delta t\gg I_{i}/\gamma_{i}^{\text{R}}$ (where $I_{i}=2m_{i}r_{i}^{2}/5$ ), while remaining small enough to prevent unphysical strand crossings (Supplementary Video SV1). The boundary particles are held fixed at their initial coordinates and are not subject to coordinate updates due to energy minimizations nor time-integrations.

**TABLE 3:** Time-integration parameters for the Brownian dynamics simulations. All simulation units are using “units real” in LAMMPS (Thompson et al., 2022).

Parameter	Symbol	Simulation units	
		Quantity	Unit
thermal energy	*k*_*B*_ *T*	6.16	Kcal/mol
DNA monomer mass	*m*_DNA_	6.18E+3	g/mol
ribosome mass	*m*_ribo_	2.11E+6	g/mol
DNA monomer rotational inertia	*I*_DNA_	7.14E+5	(g/mol)⋅Å^2^
ribosome rotational inertia	*I*_ribo_	8.45E+9	(g/mol)⋅Å^2^
dynamic viscosity	*η*	7.04E+1	(g/mol)/(fs⋅Å)
monomer translational damping	$\gamma_{\text{DNA}}^{\text{T}}$	2.39E+4	(g/mol)/fs
ribosome translational damping	$\gamma_{\text{ribo}}^{\text{T}}$	2.81E+5	(g/mol)/fs
monomer rotational damping	$\gamma_{\text{DNA}}^{\text{R}}$	9.21E+6	(g/mol)⋅Å^2^/fs
ribosome rotational damping	$\gamma_{\text{ribo}}^{\text{R}}$	1.50E+10	(g/mol)⋅Å^2^/fs
monomer translational time-scale	$\tau_{\text{DNA}}^{\text{T}}$	2.74E-1	fs
ribosome translational time-scale	$\tau_{\text{ribo}}^{\text{T}}$	1.59E+1	fs
monomer rotational time-scale	$\tau_{\text{DNA}}^{\text{R}}$	8.21E-2	fs
ribosome rotational time-scale	$\tau_{\text{ribo}}^{\text{R}}$	4.77E+0	fs
simulation timestep	Δ*t*	1.0E+5	fs

### 2.3 SMC-induced DNA loops

The 3D loop-extruding action of SMC protein complexes are simulated using the methodology of Bonato and Michieletto (Bonato and Michieletto, 2021; Ryu et al., 2021), which simulates the action of SMC heads associating with the DNA and then translocating the DNA between the head and the hinge (Nunez et al., 2019). DNA loops are created by adding harmonic bonds between “anchor” and “hinge” monomers (Figure 1D)

$$U_{\text{loop}}=k_{l}{\left(d-d_{0}\right)}^{2}\text{,}$$ (2.8)

rather than explicitly simulating the conformational changes of SMC protein complexes (Higashi et al., 2021; Nomidis et al., 2022). Due to physical considerations of the bending stiffness of dsDNA, the anchor and hinge monomers of all loops are required to be separated by a minimal loop length, *L*_min_, in units of bonded monomer distance (Figure 1D). Loops are initialized by first identifying regions of the chromosome accessible to loops by determining contiguous series of bonded monomers that are partitioned by replication forks at either end. Anchors are then randomly assigned to each of the regions with a probability proportional to the number of monomers in the region relative to the total number of looping accessible monomers across all regions. The region-assigned anchors are distributed uniformly within their respective regions. Finally, for each anchor a matching hinge is selected in a random direction along the polymer, and at a distance of bonded monomers that is equal to the minimal loop length.

Loop extrusion is simulated by periodically pausing the time-integration and updating the positions of the hinges while leaving the anchors fixed. There are two possible events during these hinge-update steps (Bonato and Michieletto, 2021) — 1) intra-strand motion in which the hinge advances in 1D along the current strand in the previously assigned direction or 2) inter-strand motion in which the hinge unbinds from the current strand with probability *p*_unbind_ and rebinds to a new strand within a 3D spherical volume centered about the anchor (Figure 1D). For this study we made the simplifying assumption that only intra-strand motion is permitted (*p*_unbind_ = 0), which has been used in other studies (Ryu et al., 2021), but the software is capable of simulating inter-strand motion. For both types of updates, only monomers whose distance from the anchor monomer is less than the grab radius, *r*_*g*_, and in the case of intra-strand updates, whose bonded monomer distance on the current strand is greater than the minimal loop length, are considered as viable update candidates (Figure 1D). The grab radius is chosen to be 50 nm based on the coiled-coil structure of SMC protein complexes (Diebold-Durand et al., 2017). Based on results showing that eukaryotic SMC complexes can traverse one-another to form Z-loops (Kim et al., 2020), we do not include any interactions between hinge and anchors that are not paired.

If the first case of intra-strand motion is selected with probability 1 − *p*_unbind_, the update monomer is selected from the set of intra-strand candidates by sampling a Poisson distribution with mean *L*_ext-avg_ and truncated at *L*_ext-max_. Based on step-size distributions measured with magnetic tweezers (Ryu et al., 2021) and analytical calculations (Takaki et al., 2021), we chose these to be *L*_ext-avg_ = 20 monomers (68 nm) and *L*_ext-max_ = 30 monomers (102 nm). Should there be no intra-strand candidates, the hinge will remain at its current monomer. If the second case of inter-strand motion is selected with *p*_unbind_, the update monomer is selected from the set of inter-strand candidates with equal probability. Should there be no inter-strand candidates following an unbinding, the hinge will remain unbound until there are inter-strand candidates in a subsequent hinge update step. The pseudocode for this process is presented in Supplementary Algorithm S1.

The length-scale of the grab radius is much greater than that of pairwise interactions between non-bonded DNA monomers, we therefore make the simplifying assumption that the DNA monomers available as hinge update candidates have a nearly uniform distribution within the spherical volume of radius *r*_*g*_ centered about any anchor. Under such conditions, the average separation distance, $\bar{d}$ , between the anchor and hinge following a hinge update may then be calculated as

$$\bar{d}=\frac{\int_{0}^{r_{g}}dr\left(r\times 4\pi r^{2}\right)}{\int_{0}^{r_{g}}dr4\pi r^{2}}=\frac{3}{4}r_{g}\text{,}$$ (2.9)

the loop will then perform on average the amount of work, ${\bar{W}}_{\text{loop}}$ , necessary to pull the hinge and anchor to their equilibrium separation distance

$${\bar{W}}_{\text{loop}}=-\left[U_{\text{loop}}\left(d_{0}\right)-U_{\text{loop}}\left(\bar{d}\right)\right]=k_{l}{\left(\bar{d}-d_{0}\right)}^{2}\text{.}$$ (2.10)

given that each extrusion event (emulated by hinge updates and subsequent pulling in this case) was measured to complete approximately 4*k*_*B*_ *T* of work (Ryu et al., 2021) and ATP hydolysis is sufficient to provide this, we estimate the spring constant in our model to be

$$k_{l}=\frac{4k_{B}T}{{\left(\bar{d}-d_{0}\right)}^{2}}\text{.}$$ (2.11)

all spatial, energetic, and probabilistic parameters for the loop-extrusion model are listed presented in Table 4.

**TABLE 4:** Energetic, spatial, and probabilistic parameters for SMC loops. All simulation units are using “units real” in LAMMPS (Thompson et al., 2022).

Parameter	Symbol	Simulation units	
		Quantity	Unit
equilibrium bond distance	*d*_0_	4*r*_DNA_	Å
grab radius	*r*_*g*_	500.0	Å
average grab distance	$\bar{d}$	3*r*_*g*_/4	Å
spring constant	*k*_*l*_	2.61E-2	Kcal/(mol⋅Å^2^)
minimum loop length	*L*_min_	5	# monomers
average 1D extrusion length	*L*_ext-avg_	20	# monomers
maximum 1D extrusion length	*L*_ext-max_	30	# monomers
unbinding probability	*p*_unbind_	0.0	n.d.

### 2.4 Polymer model simulation protocols

#### 2.4.1 Simulation software

All polymer model simulations were performed using the C++ program btree_chromo (Supplementary Table S1), which implements the binary tree model of replication states, replication within the chromosome system using the train-track model, and Brownian dynamics simulations that include the effects of SMC complexes and topoisomerases by calling LAMMPS as a library (Thompson et al., 2022). This program is executed from the command-line and takes a single input script of program directives that it then parses into commands and parameters before executing in sequence. Additionally, a number of metacommands are included that allow for sections of the script to be repeated within loops and other similar functions that aid in defining simulation protocols. All directives are documented within the project’s repository (Supplementary Table S1). Spatial, energetic, and temporal parameters for the model and subroutines that are regularly performed during the course of simulations are stored within a separate directory as a set of LAMMPS input scripts that are fed into the LAMMPS simulation object. The directory containing LAMMPS input scripts can be redefined, allowing the user to systematically test alternate chromosome models or change models on-the-fly within a simulation. Walltimes for a representative selection of the simulations presented in this study are included in Supplementary Table S2.

#### 2.4.2 Generating initial conditions

Initial configurations of the chromosome are generated using an algorithm based on a midpoint-displacement approach (Fournier et al., 1982) that builds three-dimensional, closed curves resembling Koch curves (von Koch, 1904) out of spherocylinder segments (i.e., cylinders with hemispherical caps) that overlap about the centerpoint of the caps (Supplementary Figure S1A). Given a spherical cell containing a known spatial distribution of ribosomes, the initially unrelaxed configuration of the continuum model is placed within the confines of the spherical cell by growing a circular and self-avoiding chain of spherocylinders. The freely-jointed chain of spherocylinders uses a series of decreasing cylinder lengths during the growth process to generate a chromosome configuration organized as a fractal globule (Lua et al., 2004) with clearly-defined chromosomal territories (Lieberman-Aiden et al., 2009; Sanborn et al., 2015), which is consistent with our previous lattice methodology (Gilbert et al., 2021). This is accomplished using an iterative procedure in which a specified number of spherocylinder segments are added. Self- and ribosome-avoidance are imposed at every stage between the spherocylinder segments and the spherical ribosomes. Furthermore, tracking the crossing of the spherocylinders during segment addition steps was used to prevent the introduction of knots. In the final step, spherical monomers with radii equal to the spherocylinder radii (17.0 Å) are then interpolated along the spherocylinders and any remaining monomers are inserted using an equivalent midpoint-displacement method. The model of an unreplicated Syn3A chromosome is comprised of 54,338 monomers, each containing 10 bp. This method creates suitable chromosome configurations for both the small and large Syn3A cell geometries and ribosome distributions reconstructed from cryo-ET (Gilbert et al., 2021) (Supplementary Figures S1B–C) and has been further extended to fill cell geometries with multiple circular chromosomes simultaneously (Supplementary Figure S2).

#### 2.4.3 Standard polymer model simulations

At the start of any polymer model simulation and before any Brownian dynamics steps are taken, potential particle overlaps are relaxed by running the following sequence of minimizations and short runs (Table 5): 1) minimize_soft_harmonic, 2) run_soft_harmonic, 3) minimize_hard_harmonic, 4) run_hard_harmonic, and 5) minimize_hard_FENE. The stopping criteria and maximum number of iterations for each of these are defined within the directory of input scripts. This is sufficient to relax the initial structure without significantly altering it, while remaining tolerant to the insertion of new monomers, ribosomes, or reshaping of the boundary. Brownian dynamics integration then proceeds using run_hard_FENE to simulate the system with stretching, bending, and twisting of the dsDNA polymer while preventing strand-crossings. Following replication using the train-track model (Figure 2B), the system is relaxed using the previously mentioned protocol to resolve particle overlaps that may have resulted from the addition of new monomers.

**TABLE 5:** Models used during energy minimizations (minimize_“bonds_pairs”) and Brownian dynamics time-integrations (run_“bonds_pairs”) of the system. Hard-pair interactions are used between boundary particles and all other particles for every model.

Model	DNA bonds	Pair interactions		
		DNA-DNA	DNA-ribo	ribo-ribo
soft_harmonic	harmonic	soft	soft	soft
soft_FENE	FENE	soft	soft	soft
hard_harmonic	harmonic	hard	hard	hard
hard_FENE	FENE	hard	hard	hard
topoDNA_harmonic	harmonic	topo	hard	hard
topoDNA_FENE	FENE	topo	hard	hard

#### 2.4.4 Simulations with SMC-looping and topoisomerases

Given that SMC complexes and topoisomerases were identified to be essential in Syn3A by transposon mutagenesis, we developed a simulation method to describe their interaction with the DNA at the scale of the full chromosome. Simulations of systems that include SMC-looping and the action of topoisomerases are performed using an algorithm that iteratively alternates between updating loop locations, minimizing the now non-equilibrium system’s energy, and performing Brownian dynamics steps (Supplementary Algorithm S2). We chose to use this approach because the small timesteps (Δ*t* = 0.1 ns) used to prevent strand-crossings of the 10 bp monomers would otherwise prevent us from running Brownian dynamics over timescales required for multiple loop-extrusion steps that occur on the order of seconds (Ryu et al., 2021). Intermittently, this process is stopped to run a set of Brownian dynamics steps with DNA-DNA pair interactions replaced by soft potentials permitting strand-crossings, run_topoDNA_FENE (Table 5). This and similar approaches have been used in previous studies to model the net effect of topoisomerases (Goloborodko et al., 2016a; Mitra et al., 2022b). We note that this better emulates topo-IV rather than gyrase, but we feel this is appropriate given that topo-IV is known to primarily decatenane replication products (Zechiedrich et al., 1997; Cebrián et al., 2015). The number of loops, duration of loop simulations before updates (Δ*t*_loops_), frequency of topoisomerase runs (*T*_topo_), and duration of topoisomerase runs (Δ*T*_loops_) are specified by the user. For the simulations in this study we used the following values in units of timesteps: Δ*t*_loops_ = 10,000, *T*_topo_ = 50,000, Δ*t*_loops_ = 50,000. Additionally, this algorithm was restarted every 100,000 timesteps to sample new locations for the loop anchors. Simulations show that increased loop numbers lead to greater chromosome compaction (Figure 1E), with 100 loops reducing the windowed radius of gyration by approximately 35% relative to the case with 0 loops.

### 2.5 Replication states

Beyond the configurational state of the chromosome, we wish to consider the replication state of the chromosome system. We will use a binary tree model (Taylor and Garnier, 2009) (Figure 2A), where the replication state is described by the extent of replication for each of the possible *Ori*s. The *Ori*s are labeled by their lineage relative to the mother chromosome (*m*), i.e., the root of the tree. For example, replication of the mother chromosome produces two new daughter *Ori*s, a left daughter (*ml*) and a right daughter (*mr*). This pattern continues for subsequent generations, i.e., the mother’s right daughter (*mr*) will create the daughter *Ori*s labeled *mrl* and *mrr* when it undergoes replication (Figure 2A). Aside from the initial mother chromosome, we uniformly refer to *Ori*s represented as leaves in the binary tree (Figure 2A) as “daughters” and use the label to describe the generation, i.e., a daughter (*ml*) vs. a granddaughter (*mrl*).

If we assume the mother is the zero-th generation, we can write the space of labels for the *q*-th generation as ***I***_*q*_ = {*I*_0_, *I*_1_, …, *I*_*q*−1_, *I*_*q*_}, where *I*_0_ = *m* and *I*_*j*_ ∈ (*l*, *r*) for all *j* > 0. This is essentially a *q*-dimensional vector of binary values (the zero-th element is trivially constant), but for clarity we will write it as a list of labels selecting the left/right daughters at each generation. If we have a chromosome in the *q*-th generation with the label ***i***_*q*_, then we denote the labels of its daughters in the (*q* + 1)-th generation as ***i***_*ql*_ and ***i***_*qr*_. Conversely, if we have a chromosome in the *q*-th generation with label ***i***_*q*_, then we denote the label of its mother in the (*q* − 1)-th generation as $i_{q^{\left(-\right)}}$ .

The genomic content of any daughter chromosome is determined by the extent of replication of its mother. i.e., the genomic content of the chromosome labeled ***i***_*q*_ is determined by the extent of replication, *ρ*, of the chromosome labeled $i_{q^{\left(-\right)}}$ . Given this, the replication microstate of some general chromosome system with a maximum generation of *q* is given by the vector

$$\begin{array}{cc} \rho_{q} & =\left\{\rho_{i_{0}}^{cw},\rho_{i_{0}}^{\mathrm{ccw}},\right.\\ & \left.\rho_{i_{0l}}^{cw},\rho_{i_{0l}}^{\mathrm{ccw}},\rho_{i_{0r}}^{cw},\rho_{i_{0r}}^{\mathrm{ccw}},\right.\\ & \left.\rho_{i_{1l}}^{cw},\rho_{i_{1l}}^{\mathrm{ccw}},\rho_{i_{1r}}^{cw},\rho_{i_{1r}}^{\mathrm{ccw}},\right.\\ & \left.\;\vdots \right.\\ & \left.\rho_{i_{\left(q-1\right)l}}^{cw},\rho_{i_{\left(q-1\right)l}}^{\mathrm{ccw}},\rho_{i_{\left(q-1\right)r}}^{cw},\rho_{i_{\left(q-1\right)r}}^{\mathrm{ccw}}\right\}, \end{array}$$ (2.12)

where $\rho_{i}^{cw}$ and $\rho_{i}^{\mathrm{ccw}}$ denote the extent of replication in the clockwise and counter-clockwise directions, respectively, of the chromosome with the label ***i***. For example, replication state 2 in Figure 2A is a replicating chromosome with replication proceeding from the *Ori* to the *Ter* in both clockwise and counter-clockwise directions. For notational convenience, it is assumed that ***i***_*q*_ includes all variations of labels in the *q*-th generation. For example, ***i***_2_ includes {*mll*, *mlr*, *mrl*, *mrr*} and ***i***_2*l*_ includes all 4 possible left daughters originating from the chromosomes with these labels. The number of dimensions of ***ρ***_*q*_ increases geometrically as a function of the number of considered generations as 2^*q*^. We purposefully neglect to include the terms for replication extents deeper in the binary tree that are trivially zero.

The replication microstates are subject to two constraints. First, the extent of replication of the daughter chromosome with label ***i***_*s*_ may not exceed that of its mother with label $i_{s^{\left(-\right)}}$ , i.e.,

$$\rho_{i_{s}}^{cw}<\rho_{i_{s^{\left(-\right)}}}^{cw}\;\text{and}\;\rho_{i_{s}}^{\mathrm{ccw}}<\rho_{i_{s^{\left(-\right)}}}^{\mathrm{ccw}}\text{.}$$ (2.13)

this constraint is included because it is physically impossible for a daughter to replicate DNA sequences that do not yet exist. Second, the total replication extent, $\rho_{i_{s}}$ , must be less than or equal to the total genomic content of the chromosome, i.e.,

$$\rho_{i}=\rho_{i}^{cw}+\rho_{i}^{\mathrm{ccw}}\leq 1\text{.}$$ (2.14)

these two constraints guarantee that only physically realistic replication states are permitted by the model. A change in replication microstate is denoted as

$$\Delta \rho =\left\{\Delta \rho_{i}^{cw}=a,\Delta \rho_{i}^{\mathrm{ccw}}=b,\Delta \rho_{j}^{cw}=c,\Delta \rho_{j}^{\mathrm{ccw}}=d,\ldots \right\}\text{,}$$ (2.15)

where only the forks with a nonzero change are included. Changes that lead to replication states not satisfying the two constraints are instead completed up to the maximum extent at which the constraints are still satisified.

We have previously presented a formal definition of replication microstates, we now turn to characterizing replication macrostates using state variables that correspond to experimental measurements. We begin this by defining a number of quantities that are measurable by experiments for the replication microstates. The total DNA content of a replication microstate relative to the DNA content of a single, unreplicated chromosome is given by

$$G\left(\rho_{q}\right)=1+\overset{q-1}{\underset{p=0}{\sum }}\underset{i\in I_{p}}{\sum }\left(\rho_{i}\right)$$ (2.16)

and corresponds to experimental measurements of the DNA content, such as fluorescent intensity of stained DNA. The number of *Ori*s in a replication microstate is given by

$$N_{\text{Ori}}\left(\rho_{q}\right)=1+\overset{q-1}{\underset{p=0}{\sum }}\underset{i\in I_{p}}{\sum }\Theta \left(\rho_{i}^{cw}+\rho_{i}^{\mathrm{ccw}}\right)\text{,}$$ (2.17)

where Θ is again the Heaviside step-function. The number of *Ter*s in a replication microstate is given by

$$N_{\text{Ter}}\left(\rho_{q}\right)=1+\overset{q-1}{\underset{p=0}{\sum }}\underset{i\in I_{p}}{\sum }\left[\Theta \left(\rho_{i}^{cw}-1/2\right)+\Theta \left(\rho_{i}^{\mathrm{ccw}}-1/2\right)\right]\text{,}$$ (2.18)

the ratio of the most-replicated region to the least-replicated region in a replication microstate is the number of *Ori*s divided by the number of *Ter*s and is given by

$$ϒ\left(\rho_{q}\right)=N_{\text{Ori}}\left(\rho_{q}\right)/N_{\text{Ter}}\left(\rho_{q}\right)$$ (2.19)

and corresponds to experimental measurements comparing the relative quantities of target sequences, such as qPCR. Given experimental measurements of the DNA content, *G*_exp._ and ϒ_exp._, in a population of cells, and a maximum possible generation, *p*, we wish to determine the distribution of replication microstates, P(ρ) , whose ensemble averages ( $\left\langle G\right\rangle$ and $\left\langle ϒ\right\rangle$ ) match these experimental constraints. In other words, find P(ρ) such that

$$1=\left\langle 1\right\rangle =\underset{\left\{\rho \right\}}{\sum }P\left(\rho \right)$$ (2.20)

$$G_{\text{exp}}=\left\langle G\right\rangle =\underset{\left\{\rho \right\}}{\sum }G\left(\rho \right)P\left(\rho \right)$$ (2.21)

$${ϒ}_{\text{exp}}=\left\langle ϒ\right\rangle =\underset{\left\{\rho \right\}}{\sum }ϒ\left(\rho \right)P\left(\rho \right)$$ (2.22)

are satisfied.

### 2.6 Train-track model of replication

In the “train-track” model of bacterial DNA replication (Gogou et al., 2021), the replisomes are thought to independently traverse the opposite arms of the mother chromosome while replicating the DNA (Dingman, 1974). Recent work has provided additional evidence for the train-track model by imaging independently moving replisomes using fluorescently labeled *β*-clamps (DnaN) in *E. coli* cells with synchronized replication initiation (Japaridze et al., 2020). We assume that DNA replication in Syn3A obeys the train-track model due to the aforementioned experimental evidence and the absence of multi-protein regulatory systems coded for in the minimized genome (Breuer et al., 2019; Thornburg et al., 2022).

In our implementation of the train-track model, monomers are added to the left and right daughter chromosomes following replication events by creating pairs of additional monomers centered about the location of the mother’s corresponding monomer (Figure 2B). For convenience, we will denote the spatial coordinates of the *i*-th monomer of mother, left daughter, and right daughter as $x_{i}^{m}$ , $x_{i}^{l}$ , and $x_{i}^{r}$ , respectively, and similarly denote the orientation quaternions as $q_{i}^{m}$ , $q_{i}^{l}$ , and $q_{i}^{r}$ . The coordinates of the newly-replicated left and right daughter monomers are

$$x_{i}^{l}=x_{i}^{m}+r_{\text{DNA}}\left[q_{i}^{m}{\hat{e}}_{y}{\left(q_{i}^{m}\right)}^{-1}\right]\;\text{and}\;x_{i}^{r}=x_{i}^{m}-r_{\text{DNA}}\left[q_{i}^{m}{\hat{e}}_{y}{\left(q_{i}^{m}\right)}^{-1}\right]\text{,}$$ (2.23)

where ${\hat{e}}_{y}$ is a quaternion whose scalar component is zero and vector component is the unit basis vector in the *y*-direction. The orientations of the newly-replicated left and right daughter monomers are

$$q_{i}^{l}=q_{i}^{m}\;\text{and}\;q_{i}^{r}=q_{i}^{m}\text{.}$$ (2.24)

This method is applicable to nontrivial replication states (Figure 2A), efficiently replicates the chromosome in crowded environments (Figure 2B), and can occur mid-simulation (Supplementary Video SV2). Additionally, because this method is based on the binary tree model, it can be applied for replication events involving multiple forks (e.g., $\Delta \rho =\left\{\Delta \rho_{m}^{cw}=10,\Delta \rho_{m}^{\mathrm{ccw}}=10,\Delta \rho_{ml}^{cw}=5,\Delta \rho_{ml}^{cw}=5\right\}$ ) by hierarchically replicating the new monomers. The number of monomers that will be replicated can range from 0 up to the number of monomers of unreplicated DNA along the mother chromosome.

For the purposes of this model, we neglect to include the difference in the leading-versus lagging-strands, and model the fork itself as a standard DNA monomer. We add a harmonic angle potential of the form

$$U_{\text{fork}}^{b}=k_{\text{fork}}{\left(\theta -\theta_{0}\right)}^{2}$$ (2.25)

between the following triplets of particles formed by the fork (f) and three bonded monomers, mother (m), left (l), and right (r): (m-f-l), (m-f-r), and (l-f-r). The parameters are *θ*_0_ = 2*π*/3 radians and *k*_fork_ × (1 radian)^2^ = *κ*_*b*_. Additionally, there are no torsional interactions between (f-m), (f-r), or (f-l).

### 2.7 Chromosome segregation calculations

Given a pair of replication forks producing left and right daughters, each of which may themselves be potentially undergoing replication, the sets of *N*_*l*_ and *N*_*r*_ replicated monomers belonging to the left and right daughters are $\left\{x_{i}^{l}\right\}$ and $\left\{x_{i}^{r}\right\}$ , respectively. For example, in state 4 shown in Figure 2A, the daughter sizes are *N*_*l*_ = 60 and *N*_*r*_ = 90 for fork *m* and *N*_*l*_ = 30 and *N*_*r*_ = 30 for fork *mr*. Segregation of the daughter chromosomes can be investigated using these sets of coordinates for all pairs of forks in a system with a nontrivial replication state by analyzing the disentanglement and the partitioning.

#### 2.7.1 Disentanglement

The number of monomers belonging to the same (s) daughter within a radius, *R*, of the *i*-th replicated monomer of the left/right (l/r) daughter are

$$n_{i}^{s,\left(l/r\right)}\left(R\right)=\overset{N_{\left(l/r\right)}}{\underset{j=1,j\neq i}{\sum }}\Theta \left(R-\left|x_{i}^{\left(l/r\right)}\right.-\left.x_{j}^{\left(l/r\right)}\right|\right)$$ (2.26)

and the number of monomers belonging to the opposite (o) daughter within that radius are

$$n_{i}^{o,\left(l/r\right)}\left(R\right)=\overset{N_{\left(r/l\right)}}{\underset{j=1}{\sum }}\Theta \left(R-\left|x_{i}^{\left(l/r\right)}\right.-\left.x_{j}^{\left(r/l\right)}\right|\right)\text{.}$$ (2.27)

the fraction of monomers on the same daughter within the radius about the *i*-th monomer is

$$\varphi_{i}^{\left(l/r\right)}\left(R\right)=\frac{n_{i}^{s,\left(l/r\right)}\left(R\right)}{n_{i}^{s,\left(l/r\right)}\left(R\right)+\left[N_{\left(l/r\right)}/N_{\left(r/l\right)}\right]\times n_{i}^{o,\left(l/r\right)}\left(R\right)}\text{,}$$ (2.28)

the average fraction for each daughter is

$${\bar{\varphi }}^{\left(l/r\right)}\left(R\right)=\frac{1}{N_{\left(l/r\right)}}\overset{N_{\left(l/r\right)}}{\underset{i=1}{\sum }}\varphi_{i}^{\left(l/r\right)}\left(R\right)\text{,}$$ (2.29)

and the degree of disentanglement (DoD) is a function of these

$$\text{DoD}\left(R\right)=f\left({\bar{\varphi }}^{l}\left(R\right),{\bar{\varphi }}^{r}\left(R\right)\right)\text{.}$$ (2.30)

we use the harmonic mean for this function as it provides a conservative estimate, then shift and scale the result such that the range of the degree of disentanglement is [0,1]

$$f\left({\bar{\varphi }}^{l}\left(R\right),{\bar{\varphi }}^{r}\left(R\right)\right)=2\times \left(\frac{2\times \left[{\bar{\varphi }}^{l}\left(R\right)\times {\bar{\varphi }}^{r}\left(R\right)\right]}{{\bar{\varphi }}^{l}\left(R\right)+{\bar{\varphi }}^{r}\left(R\right)}-\frac{1}{2}\right)\text{.}$$ (2.31)

using this definition, 0 corresponds to a fully entangled system that overlaps everywhere and 1 corresponds to a disentangled system whose constituent parts are separated by at least a distance *R*. When calculating the DoD for our system we use *R* = 4*r*_DNA_.

#### 2.7.2 Partitioning

We evaluate the extent to which the daughter chromosomes are partitioned by calculating the distance between their centers of mass (CoM)

$$d_{\text{CoM}}=|X_{\text{CoM}}^{l}-X_{\text{CoM}}^{r}|\text{,}$$ (2.32)

where

$$X_{\text{CoM}}^{\left(l/r\right)}=\frac{1}{N_{\left(l/r\right)}}\overset{N_{\left(l/r\right)}}{\underset{i=1}{\sum }}x_{i}^{\left(l/r\right)}\text{.}$$ (2.33)

the *d*_CoM_ was then compared to a length-scale characteristic of what we will refer to as an “ideal partitioning”. In an ideal partitioning, we assume the daughters will occupy volumes that are proportional to their relative sizes, *N*_*l*_ and *N*_*r*_, in units of monomers and share a planar interface with minimal surface area. Given a radius of the spherical confinement, *r*, we then determine the distance between their centers of mass in this ideal scenario, which we will refer to as *L*_partition_(*N*_*l*_, *N*_*r*_, *r*) (Supplementary Material).

### 2.8 Intra- and inter-daughter contact calculations

While the interactions between equivalent loci on daughter chromosomes are distinguishable in the *in silico* model, they are indistinguishable to most sequence-based experimental techniques, such as 3C methods. However, efforts have been made to resolve these interactions in eukaryotic systems with sister-chromatid-sensitive Hi-C (Mitter et al., 2020) and bacterial systems with recombinase assays (Lesterlin et al., 2012; Espinosa et al., 2020; Oomen et al., 2020). We extend our methodology for *in silico* contact maps (Supplementary Material) to the case of replicating chromosomes by using the relative position within the bond topology (Figure 2A) of the monomer identified as the *Ori* to determine equivalent loci containing identical DNA sequences on daughter chromosomes (Figure 3A). If F is the true contact map encoding the entirety of all intra- and inter-daughter interactions, then we will denote the sequence-equivalent map reflecting 3C observations as $\tilde{F}$ . The sequence-equivalent map is determined by summing the contributions for each of the possible interactions (Figure 3B) before rebalancing the resulting matrix. Additionally, this methodology can be further extended to address the determination of *in silico* contact maps that represent a mixture of chromosomes in different replication states (Supplementary Figures S6, S7) by calculating weighted averages of sequence-equivalent maps, which outside of isolated cases (Nagano et al., 2013; Ramani et al., 2017; Kos et al., 2021), are what 3C libraries are ultimately measuring within a population of unsynchronized cells.

### 2.9 Martini model preparation

Simulating a Martini model of the Syn3A chromosome requires CG starting coordinates and a CG topology that specifies all the bonded and non-bonded interactions of the DNA model (Uusitalo et al., 2015). In traditional protocols, both are generated by forward mapping an all-atom structure to Martini resolution (Uusitalo et al., 2015; 2017; Kroon et al., 2022). However, given the size of the chromosome, this approach becomes infeasible. Thus we follow a strategy that splits the generation of topology and coordinates into two separate steps. First, we generate starting coordinates at Martini resolution directly from the polymer model’s coordinates using a new backmapping protocol. In the second step, the chromosome topology is generated from the genome sequence. Both steps are implemented in a Python package, Polyply, which focuses on facilitating the setup of MD simulation of complex polymer systems (Grünewald et al., 2022).

#### 2.9.1 Generation of the starting coordinates

**FIGURE 4:**
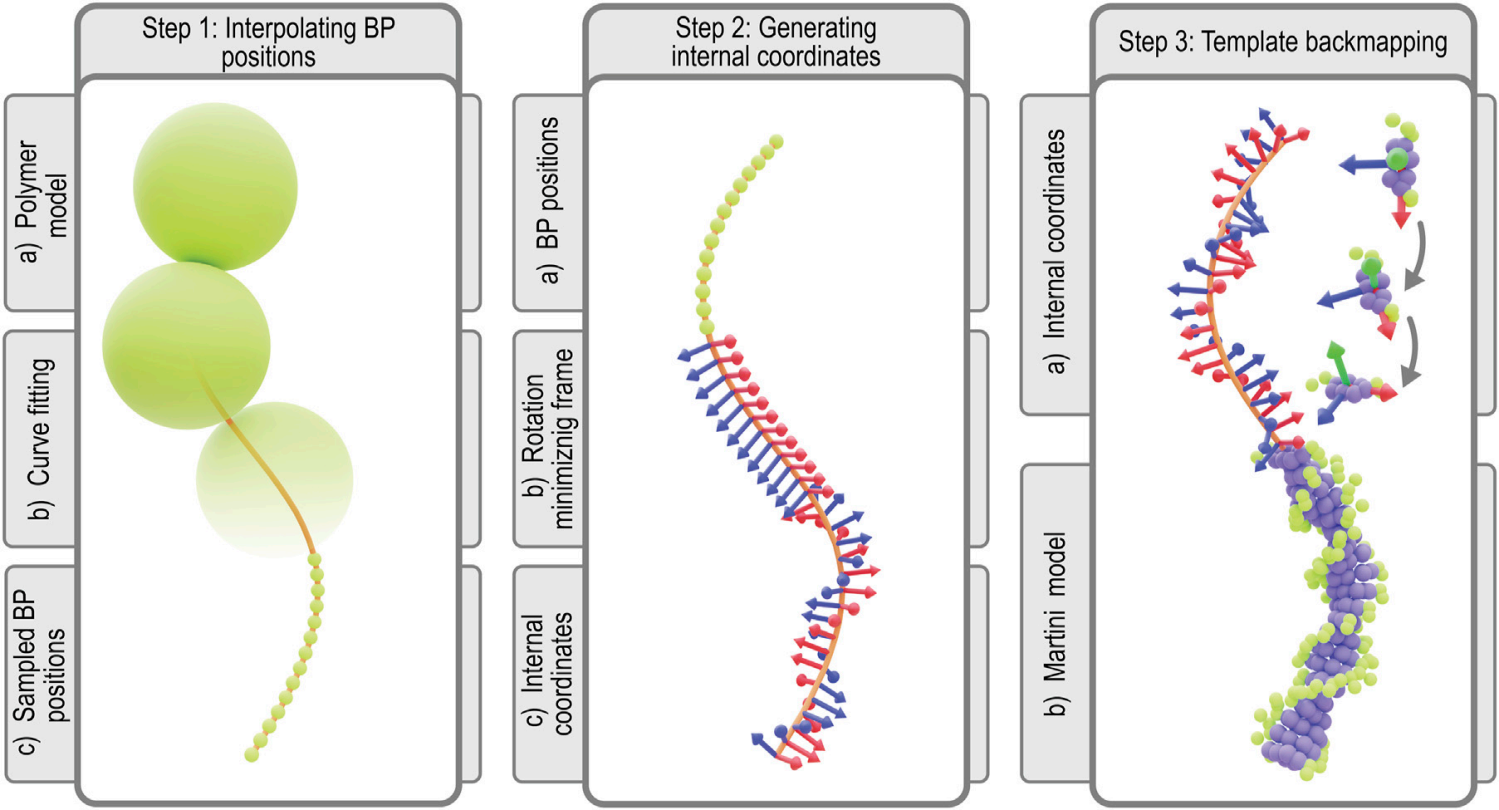
Martini backmapping protocol: Schematic depicting the steps in the protocol used to generate coordinates in the Martini representation. By backmapping a dsDNA polymer model, the protocol efficiently creates a near-atomistic model of the entire chromosome. In the final output Martini model at the far-right, each bp is represented by 7 purple beads for the nucleobases (3 per pyrimidine, 4 per purine) and 3 green beads for each backbone (2 per sugar, 1 per phosphate), for a total of 13 Martini beads per bp (Uusitalo et al., 2015).

The protocol for constructing coordinates for the chromosome at Martini resolution starts with interpolating the 10 bp per monomer polymer model generated as previously described (Figure 4, step 1). To this end, a periodic B-spline, ***m*** (*s*), is fitted to the monomer positions, {***x***_*i*_}, which represents the chromosome’s helical axis (Dierckx, 1996; Virtanen et al., 2020). Along the helical axis, the bp positions, {***m***_*j*_}, are sampled such that each segment of the curve between monomer centers contains 10 bp spaced equidistantly. Next, we align bp template coordinates at the Martini level using the resulting bp positions. To properly align the templates, we have to define the internal coordinates $\left({\hat{u}}_{j},{\hat{f}}_{j},{\hat{v}}_{j}\right)$ for all the sampled positions (Figure 4, step 2).

In order to construct these internal coordinates, we use a rotation minimizing frame (RMF). An RMF is a reference frame that does not rotate around the instantaneous tangent of the curve ***m*** (*s*), which is defined continuously along any B-spline. The stability of an RMF is ideal for our application since discontinuities in the orientation of consecutive bases will lead to an unrealistic chromosome geometry. The RMF is constructed along the sequence of bp positions, {***m***_*j*_}, using the double reflection method outlined by (Wang W. et al., 2008). The paper describes a simple and fast algorithm for approximating our chromosome’s RMF with a global error in the order of $O\left(h^{4}\right)$ , where *h* is the distance between consecutive bps.

To apply the double reflection method and construct the RMF, we first calculate the instantaneous tangent ${\hat{u}}_{j}$ on the bp positions using numerical differentiation. To ensure the double reflection method’s accuracy, the approximation error of the tangents ${\hat{u}}_{j}$ to the true tangent vector, ***m*** ′(*s*), must be of the order $O\left(h^{5}\right)$ . Given an arbitrary starting reference vector, the RMF can now be constructed along the entire helical axis.

In order to transform the RMF to the internal coordinates of the chromosome, we must apply two additional transformations to the RMF. Since Syn3A′s chromosome is circular, the additional boundary condition that has to be satisfied is the continuity between the first and last bp’s internal coordinates. This condition is realized by applying an additional twist per bp, i.e., a rotation over ${\hat{u}}_{i}$ , to compensate for a possible discontinuity in the RMF. Additionally, to incorporate the intrinsic helical pitch of B-DNA, an additional twist of 34.3° per bp is applied to each frame (Sinden, 1994). Approximating the DNA’s intrinsic structure by a uniformly twisting double helix is justified by the absence of NAPs in Syn3A, resulting in the chromosome not sustaining any significant supercoiling (Gilbert et al., 2021). Finally, using a rigid transformation, templates of the Martini bps are placed on the sampled positions and aligned to the corresponding internal coordinates, building the starting coordinates of the Martini chromosome model (Figure 4, step 3).

#### 2.9.2 Generation of the chromosome topology

The topology at the Martini level comprises the bead-type assignments (i.e., non-bonded interactions), the bonded interactions, and possibly structural biases such as an elastic network. The typical frameworks for generating topology files at the Martini level take an all-atom structure as input (Brooks et al., 1983; Liwo et al., 1997; Case et al., 2005; Phillips et al., 2005; de Jong et al., 2013; Machado and Pantano, 2016; Kroon et al., 2022; Abraham et al., 2023). Subsequently, a connectivity graph is generated from the distance matrix and valency-based rules. From this graph, using the all-atom to Martini correspondence defined in the mapping of the nucleobases, the Martini topology is created. This process is called resolution transformation. Using the complete all-atom connectivity graph makes procedures invariant to molecular topology and allows the identification of chemical modifications (e.g., methylation) on the fly. However, the underlying subgraph isomorphism is an NP-complete problem. Thus, while this procedure is very rigorous, it is not very efficient.

Instead, we extended the multiscale graph matching protocol implemented in Polyply to dsDNA. In essence, the protocol performs a resolution transformation from the residue graph to target resolution, in this case, Martini. Utilizing the residue graph gives the needed speed-up to handle polymers of the size of the chromosome. Even though the algorithm still uses a subgraph isomorphism, it is faster since it only works on the residue graph instead of the full molecule graph. Using this algorithm, the molecule topology is generated in two steps: 1) From a set of provided building blocks, all bonded interactions and bead-type assignments are determined for the individual nucleobases (i.e., intra-residue). 2) Bonded interactions, which span multiple residues, are assigned by finding all valid subgraph isomorphisms between graph fragments that describe these inter-residue interactions and the target graph at the residue level. For each match, the bonded interactions are added to the topology. Furthermore, the bead-types are also modified to account for the links between residues where needed. The second strand is generated in the same way by running the algorithm on the complementary single-strand sequence.

The intra- and inter-residue graph fragments, referred to as blocks and links, need to be provided to Polyply as input files. Thus we have extended the Polyply library with data files that describe DNA parameters for Martini2 (Uusitalo et al., 2015). Furthermore, for convenience, Polyply was extended with a parser for .fasta and .ig data files that describe DNA sequences. Most importantly, an automatic recognition of circular DNA is possible when provided with an .ig data file.

Finally, we note that all Martini DNA needs a secondary structure stabilization (i.e., elastic network). Informed by the generated starting coordinates of the Martini chromosome, an elastic network connects nearby beads with harmonic bonds. A simple auxiliary script was used to add the elastic network to the already existing topology generated with Polyply.

#### 2.9.3 Additional structural components

In addition to modeling the intrinsic dynamics of the chromosomal DNA, the polymer model also captures the DNA interacting with the cell membrane and ribosomes. For our Martini chromosome model, these contributions can also explicitly be taken into account with the same near-atomistic resolution. To model the ribosomes, we use a bacterial homolog previously published by (Uusitalo et al., 2017). Initially, we attempt to align the ribosomes with their counterparts in the polymer model. In this step, steric clashes with the chromosome can occur, which we resolve by applying small random rigid body transformations to the ribosomes. The translation length in this transformation acts as a fudge factor, which slowly increases per failed iteration. Lastly, a realistic cell membrane is constructed using the TS2CG tool, including both a realistic lipid composition and a representative membrane protein density (Pezeshkian et al., 2020).

## 3 Results

### 3.1 Diffusion of ribosomes and DNA monomers

The spatial heterogeneity of macromolecules and complexes within the cell and the need for them to encounter one another *via* diffusion strongly contribute to the stochastic nature of gene expression. For example, a RNA polymerase (RNAP) must diffuse to a gene to perform transcription and a mRNA and ribosome must diffuse to one another to perform translation. Some of these reactions can become coupled with one another, such as multiple ribosomes reading the same mRNA (polysomes) or a ribosome reading a nascent mRNA that is still being transcribed from a RNAP (expressomes - coupled transcription and translation) (O’Reilly et al., 2020). These couplings have been observed to varying extents in multiple bacteria. The proportion of ribosomes found in polysomes in *E. coli* has been reported as high as 80% (Bremer and Dennis, 2008), and the proportion in an organism related to Syn3A, *Mycoplasma pneumoniae*, has been reported as 26% (Xue et al., 2022). Expressomes have been observed to a lesser extent, the proportion of ribosomes participating in one only being 3% of ribosomes in *M. pneumoniae* (O’Reilly et al., 2020). Based on cryo-ET we estimated the proportion of ribosomes in polysomes in Syn3A is 25%–40% and from prior simulations we estimate the proportion of ribosomes in close enough proximity to the DNA to form an expressome to be roughly 20% (Gilbert et al., 2021). In the WS-WCM of Syn3A, polysomes were shown to be a critical factor in accurately doubling the proteome over the course of a cell cycle (Thornburg et al., 2022). Before we try to quantify how the effects of these coupled mechanisms affect the spatial organization and diffusion of the chromosome and ribosomes (Mondal et al., 2011), here we quantify how the chromosome and complete, intact ribosomes affect the diffusion of one another at the scale of a whole Syn3A cell.

**FIGURE 5:**
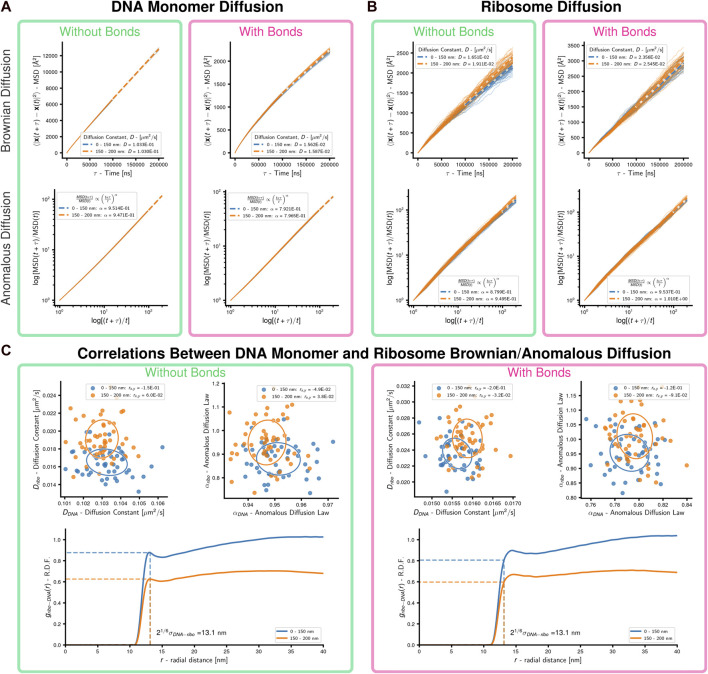
Cell-scale diffusion of ribosomes and DNA monomers: 50 replicates of a system with an unreplicated 54,338 monomer chromosome in 200 nm radius cell containing 500 uniformly distributed ribosomes were simulated. **(A)** Brownian and anomalous diffusion of DNA monomers with and without bonds forming DNA polymer. For the two concentric spherical shells, dashed lines are the least-squares fits for the Brownian diffusion constant (linear-linear) and anomalous diffusion power-law (log-log), respectively. **(B)** Brownian and anomalous diffusion of ribosomes with and without bonds forming DNA polymer. For the two concentric spherical shells, dashed lines are the least-squares fits for the Brownian diffusion constant (linear-linear) and anomalous diffusion power-law (log-log), respectively. **(C)** Correlations between DNA and ribosome diffusion with and without bonds forming DNA polymer. Above are scatter plots with covariance ellipses for Brownian and anomalous diffusion, Pearson correlation coefficients are reported in the legends. Below are estimates of the radial distribution function of DNA monomers about ribosomes, the dashed lines indicate the cutoff for WCA interactions. Results are shown for the two concentric spherical shells.

Simulations were performed on 50 replicate systems of representative Syn3A cells with a radius of 200 nm, each of which contained 500 uniformly distributed ribosomes and a randomly-generated configuration of a single unreplicated chromosome. Following an initial energy minimization of the standard polymer model of the chromosome, bond $\left(U_{i}^{s}\right)$ , bending $\left(U_{i}^{b}\right)$ , and twisting ( $U_{i}^{a}$ and $U_{i}^{t}$ ) interactions between all DNA monomers were added/removed from the system for two test cases, which we will refer to as “with bonds” and “without bonds”, respectively. We analyzed the diffusion of DNA monomers and ribosomes in two regions of the cell: 1) a central spherical volume extending to 150 nm within which surface effects are assumed to be negligible (Śmigiel et al., 2022) and 2) an outer concentric spherical shell extending from 150 nm to 200 nm. Particles are assigned to these shells using their initial coordinates at *t* = 0. Mean-squared displacements of the DNA monomers and ribosomes were calculated as ensemble averages within each of the regions for each replicate system, these are the transparent time-traces (Figures 5A, B), respectively. Least-squares fits were then used to determine the Brownian diffusion constants, *D*, and the power-law exponent, *α*, for the case of anomalous diffusion (Barkai et al., 2012; Oliveira et al., 2019; Muñoz-Gil et al., 2021) for each replicate (Supplementary Material). The ensemble-averaged values across replicates are reported in the legends (Figures 5A, B).

In the absence of bonds, the DNA monomers move following nearly Brownian diffusion. Bonding the monomers causes their motion to become sub-diffusive with *α* ≈ 0.79 for both inner and outer regions (Figure 5A). Sub-diffusive motion is an expected result for monomers within polymers, but Rouse dynamics predict *α* = 0.5 for times shorter than the relaxation time (Doi and Edwards, 1988). Our result agrees with theoretical predictions (*α* = 0.75) for short-time segmental motion in stiff worm-like chains with contour lengths much longer than their persistence length (Berg, 1979) and experimental measurements (*α* = 0.75) of large (relative to void) particle diffusion in networks of stiff filaments (Amblard et al., 1996). We repeated similar simulations using systems whose initial conditions were generated without ribosomes to probe the origin of DNA monomers’ sub-diffusive behavior in our model. In the scenario without ribosomes the DNA monomers are less sub-diffusive with *α* ≈ 0.85 (Supplementary Figure S4), which suggests sub-diffusive motion is a result of the confined chromosome forming a stiff polymer network. Our model’s deviation from observed sub-diffusive behavior (*α* = 0.4) of chromosomal loci in *E. coli* (Weber et al., 2010a) is likely a result of neglecting the viscoelastic nature of the bacterial cytoplasm (Weber et al., 2010b). These results for the DNA are observed for both the inner and outer regions.

Ribosomes move sub-diffusively within the inner region of the system without bonds and approach Brownian diffusion in the outer region of the system without bonds, where the DNA density is lower. When bonds are added to the system the ribosomes in the inner region undergo motion closer to Brownian diffusion. Comparing the radial distribution functions (Patrone and Rosch, 2017) of DNA monomers about the ribosomes (Figure 5C; Supplementary Material), we determined that this was a result of the system with bonds creating a polymer mesh with persistent voids (Sorichetti et al., 2020; Xiang et al., 2021) for the ribosomes to diffuse within, in contrast to the case without bonds where the DNA monomers rapidly diffuse and are closely crowded around the ribosomes. It should be noted that the asymptotic approach of the radial distribution functions in the outer shell approaching a value less than one is expected due to the cutoff radius including empty volumes outside the boundaries of the cell. The Brownian diffusion constants of ribosomes in systems with bonds is within the range of experimental measurements in other bacteria (Bakshi et al., 2012; Sanamrad et al., 2014). No significant correlations between the Brownian/anomalous diffusion of the DNA monomers and ribosomes were observed, as can be seen by the covariance ellipsoids and Pearson correlation coefficients reported in the legends (Figure 5C). These were not repeated for the case of chromosomes with loops and topoisomerase due to the non-equilibrium nature of those simulations.

### 3.2 Chromosome segregation

There is experimental evidence of chromosome segregation during replication (Nielsen et al., 2006), and furthermore, segregation of replicating chromosomes in nontrivial replication states in *E. coli* (Youngren et al., 2014). For the purposes of this study, we separate chromosome segregation into two effects: A) the disentanglement of daughter chromosomes and B) the partitioning of the daughter chromosomes’ centers of mass into different regions of the mother cell. Both chromosome disentanglement through the influence of compaction (Goloborodko et al., 2016b) caused by DNA-looping (Marko, 2009; 2011; Goloborodko et al., 2016a; Brahmachari and Marko, 2019) and the partitioning of chromosomes through entropic repulsion of polymer topologies within confinements (Jun and Mulder, 2006; Jung and Ha, 2010; Jung et al., 2012; Junier et al., 2013; Wasim et al., 2021; Mitra et al., 2022a) have been previously been studied in computational settings.

**FIGURE 6:**
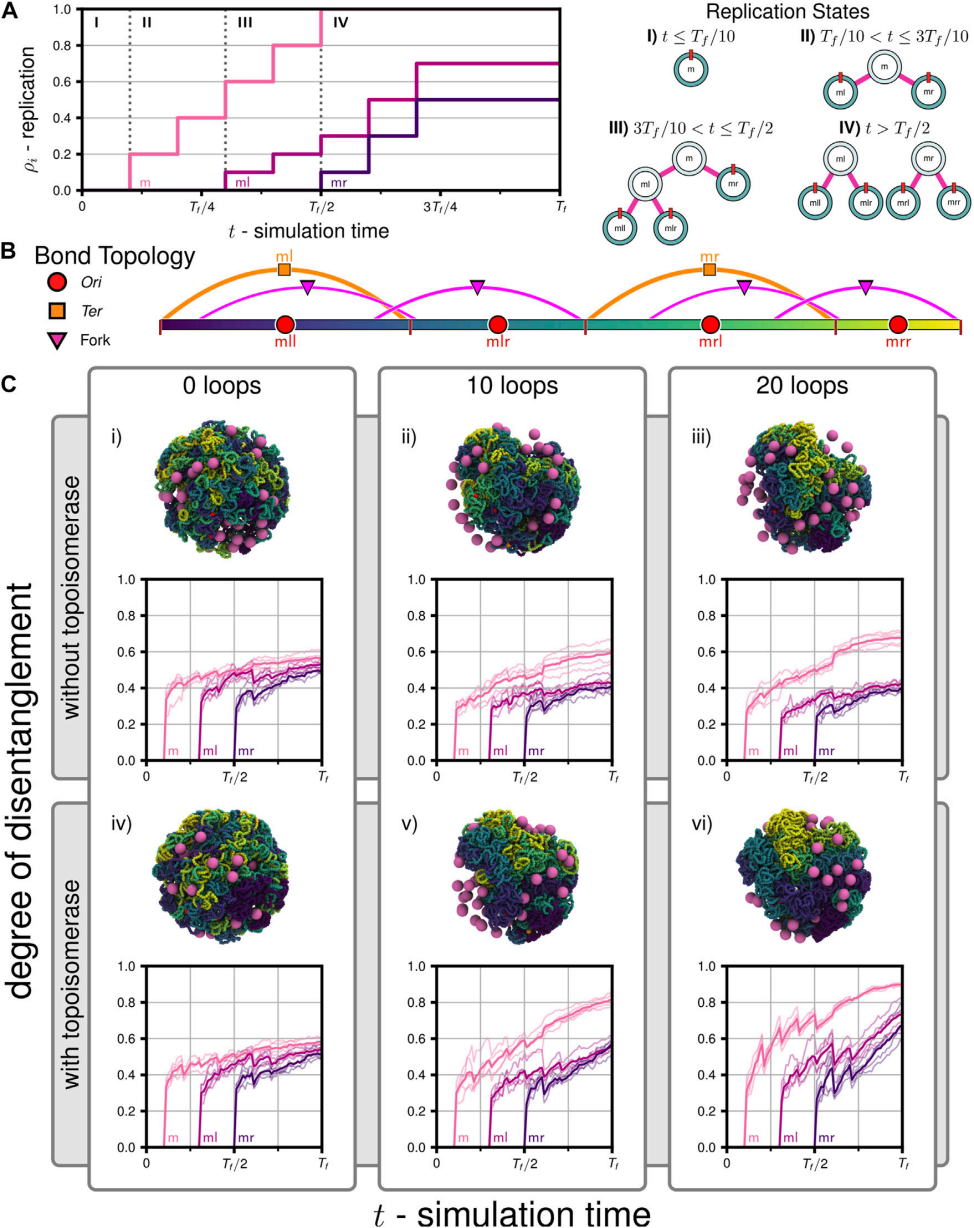
Disentanglement of daughter chromosomes during replication: **(A)** Replication progress $\left(\rho_{i}=\rho_{i}^{cw}+\rho_{i}^{\mathrm{ccw}}\right)$ as a function of time for the set of simulations testing the influence of loop extrusion and topoisomerases on disentanglement. The corresponding binary tree representations of the replication states are shown on the right. **(B)** Bond topology of the replicated system at *t* = *T*_*f*_. **(C)** Mean degree of disentanglement as a function of simulation time for the six cases (i-vi) considered (solid line), five replicate systems were simulated for each case (faint lines). The trace labeled *m* corresponds to the entanglement of *ml* and its descendents (*mll*-purple,*mlr*-blue) with *mr* and its descendents (*mrl*-green,*mrr*-yellow), *ml* corresponds to the entanglement of the replicated region of *ml*, i.e., the regions of *mll* (purple) and *mlr* (blue) connected by forks, and *ml* corresponds to the entanglement of the replicated region of *mr*, i.e., the regions of *mrl* (green) and *mrr* (yellow) connected by forks. Snapshots of the final configurations at *t* = *T*_*f*_ are shown above each plot, respectively.

We probed chromosome segregation using a toy system approximately one-tenth the volume of a Syn3A cell with similar number densities (90 nm radius, a single unreplicated 50,000 bp chromosome, and 50 ribosomes). We carried out a series of simulations to probe the essential nature of proteins hypothesized to be necessary for simultaneous chromosome segregation during replication. Over the course of the simulations, the 5,000 monomer chromosome was replicated and the *Ori* to *Ter* ratio changed in the following sequence I) 1:1, II) 2:1, III) 3:1, and IV) 4:2 (Figure 6A). The final replication state is that of two fully replicated daughter chromosomes, each of which are themselves in the process of replication (Figure 6B), where the DNA content has more than tripled to 16,000 monomers (160,000 bp). The number of loops present in the systems were varied between 0, 10, and 20, and these systems were then simulated with and without the action of topoisomerases, for a total of six cases (i-vi in Figure 6C). Five independently generated initial conditions were used to prepare five replicate simulations per case, for a total of thirty simulations. Each simulation was run until the final time of *T*_*f*_ = 2.0E+7 timesteps using the looping and topoisomerase algorithm and parameters described in Section 2.4, which corresponds to 2,000 extrusion events for each loop present in the system. At every timestep, we used the binary tree model to group monomers into left/right daughters and their descendants, each with *N*_*l*_ and *N*_*r*_ monomers, respectively, and used those groupings to analyze the disentanglement and partitioning of the daughter chromosomes about each set of replication forks (*m*,*ml*,*mr*). We have completed an equivalent proof-of-concept simulation on the full system with 54,338 monomers in a 200 nm cell containing 500 ribosomes (Supplementary Video SV2).

#### 3.2.1 Disentanglement of daughter chromosomes

We calculated a metric describing the relative number of contacts between different daugther chromosomes, which we will refer to as the degree of disentanglement, as a function of simulation time (Figure 6C) for all six cases. First, we note that for all cases the degree of disentanglement exhibits abrupt decreases when portions of the chromosome are replicated, i.e., each abrupt decrease is a result of the step-wise increases in the replication state (Figure 6A). This result was anticipated because daughter chromosomes are in close spatial proximity as they are generated using the train-track model (Figure 2B) and is consistent with experimental observations of daughter(/sister) chromosome cohesion due to precatenanes in the wake of the replication fork (Wang X. et al., 2008; Cebrián et al., 2015). This effect would be less-pronounced if a smaller fraction of the genome was replicated in each step. We find that both topoisomerase and loop-extruding SMC protein complexes are necessary for daughter chromosomes to be disentangled as replication occurs. In cases i-iii without topoisomerases, topological constraints cannot be resolved and the system remains entangled (Figure 6C). Interestingly, while adding loops in cases ii and iii assists in disentangling *ml* and *mr* about fork *m*, the presence of loops increases the entanglements of *mll* with *mlr* about fork *ml* and *mrl* with *mrr* about fork *mr*, respectively (Figure 6C). Within our model, looping in the absence of topoisomerases is deletrious for subsequent rounds of replication because enhanced compaction increases the likelihood that topological constraints are introduced during replication. However, including solely topoisomerase in case iv is not effective at disentangling the chromosome (Figure 6C). We hypothesize that this is because diffusive motion is insufficient to cross strands when the soft potential emulating topoisomerases in our model is active and that loop-extrusion assists to isolate possible strand-crossings before completing the crossings in subsequent extrusion steps to resolve topological constraints. In cases v and vi, we find the greatest degrees of disentanglement (Figure 6C). When comparing the disentanglement of *ml* and *mr* about fork *m* between cases ii-iii and v-vi, we find that a plateau is reached in cases ii-iii when the topological constraints cannot be resolved (Figure 6C). In summary, we find that systems require both topoisomerase and loops to simultaneously disentangle all daughter chromosomes as they are being replicated. Furthermore, increasing the number of loops increases the rate of disentaglement, as seen in case vi *versus* v. The trends quantified by the degree of disentanglement can also be qualitatively observed in the snapshots of the final configurations at *t* = *T*_*f*_ (Figure 6C). The degree of disentanglement was calculated for the proof-of-concept simulation of the full chromosome (Supplementary Video SV2) and shows the same behavior as the cases (v and vi) with both SMC and topoisomerase (Supplementary Figure S8).

#### 3.2.2 Partitioning of daughter chromosomes

**FIGURE 7:**
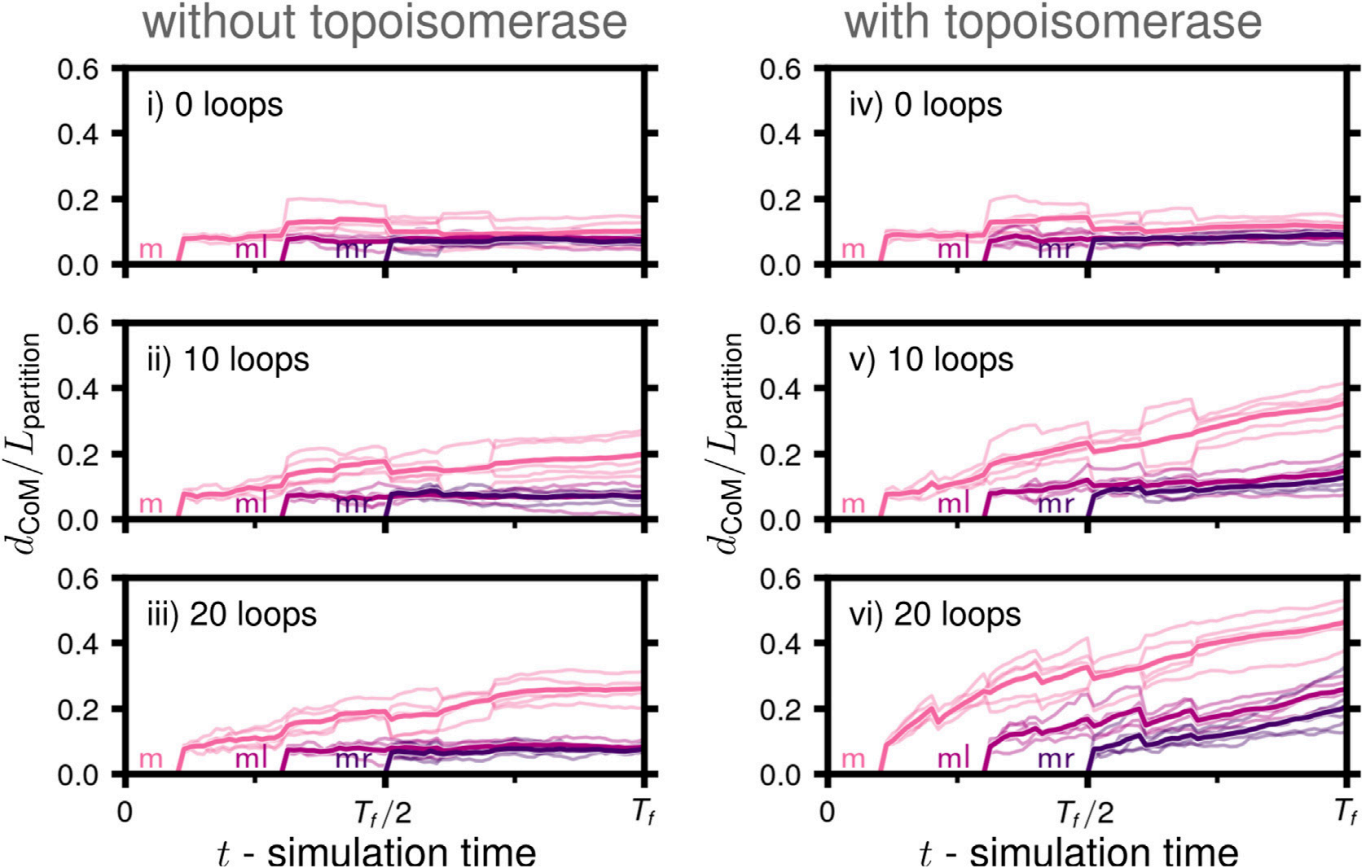
Partitioning of daughter chromosomes during replication: Mean separation of daughters’ centers of mass (*d*_CoM_) relative to the length-scale of ideal partitioning, *L*_partition_(*N*_*l*_, *N*_*r*_, *R*_sphere_), in a spherical volume of daughters with *N*_*l*_ and *N*_*r*_ monomers as a function of simulation time for the six cases (i-vi) considered (solid line), five replicate systems were simulated for each case (faint lines). The trace labeled *m* corresponds to the separation of *ml* and its descendents (*mll*,*mlr*) with *mr* and its descendents (*mrl*,*mrr*), *ml* corresponds to the separation of the replicated region of *ml*, i.e., the regions of *mll* and *mlr* connected by forks, and *ml* corresponds to the separation of the replicated region of *mr*, i.e., the regions of *mrl* and *mrr* connected by forks (Figure 6B).

We calculated the Euclidean distance separating the daughters’ centers of mass relative to an ideal partitioning, *L*_partition_(*N*_*l*_, *N*_*r*_, *R*_sphere_), to assess the extent to which the daughter chromosomes had been partitioned to different volumes within the cytoplasmic space (Figure 7). If the daughters and their descendants have an equal number of monomers (*N*_*l*_ = *N*_*r*_), ideal partitioning would correspond to them occupying identical hemispherical volumes (Supplementary Figure S2). The daughters’ centers of mass would then be found at the centroids of the hemispheres and separated by 3*R*_sphere_/4. The functional dependence of the ideal partitioning on *N*_*l*_ and *N*_*r*_ accounts for possible asymmetries in nontrivial replication states, such as states III and IV (Figure 6A). Similar to the results of the degree of disentanglement (Figure 6C), we find that partitioning was the most complete in case vi with topoisomerase and the greatest number of loops (Figure 7). However, over the timescales simulated, the distance separating the daughters’ centers of mass is still relatively insignificant as compared to the size of the confining volume. This can be observed qualitatively in the manner in which the compacted globules of the disentangled daughters are folded around one another (Figure 6C). Based on this, we conclude that disentanglement is necessary for partitioning to occur, and due to the necessity of topoisomerase and loops for disentanglement, successful partitioning is also dependent on topoisomerase and loops. However, absent a regulatory system introducing a spatial heterogeneity or active force, the partitioning in our model proceeds over a much longer time-scale than the disentanglement. This can be seen in case vi, where the degree of disentanglement about fork *m* is reaching a plateau near one, indicating that all that remains is an interface between the now disentangled daughters (Figure 6C), while the extent of partitioning has yet to reach half of the ideal distance, *L*_partition_ (Figure 7).

#### 3.2.3 Contact maps between daughter chromosomes

**FIGURE 8:**
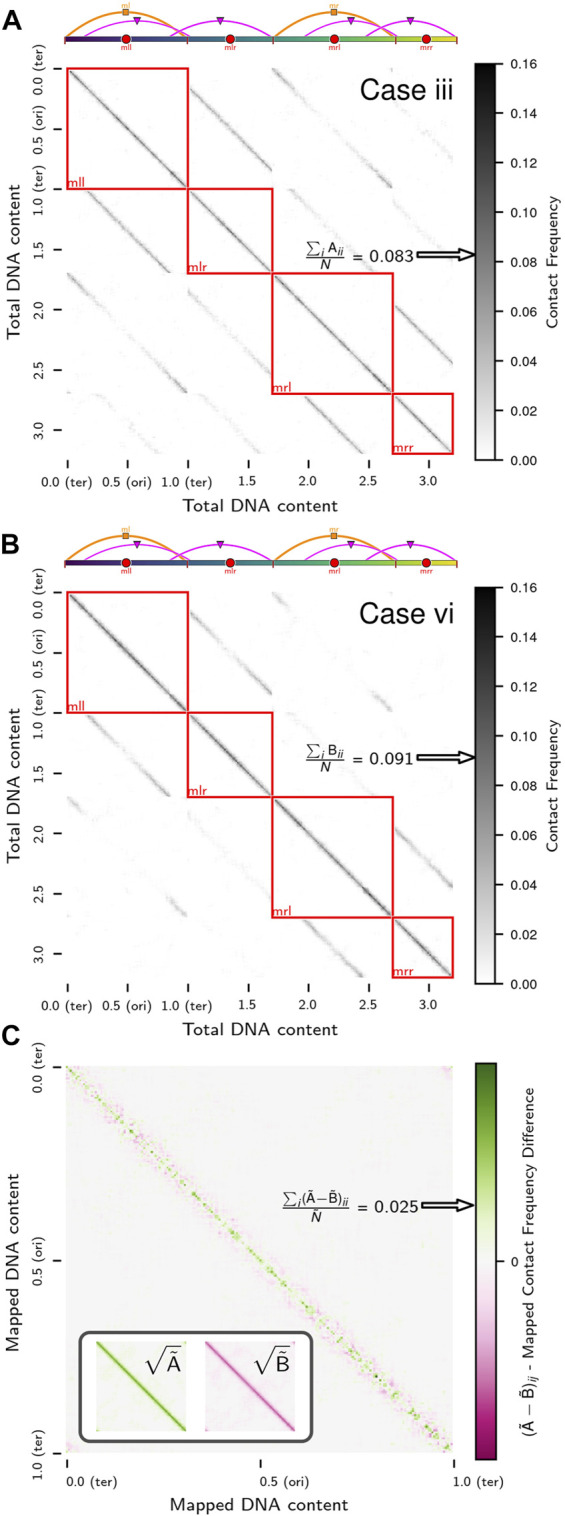
Contact maps of replicating chromosomes: **(A)** True contact map, A , for case iii (20 loops, without topoisomerase) of replicating chromosome system in Figure 6C. **(B)** True contact map, B , for case vi (20 loops, with topoisomerase) of replicating chromosome system in Figure 6C. **(C)** Difference in sequence-equivalent contact maps $\tilde{A}$ and $\tilde{B}$ , which are created by mapping A and B , respectively, using the procedure illustrated in Figure 3B. Inset are the sequence-equivalent maps, displayed after taking an element-wise square-root to enhance visual clarity. Within A-C, the arrows along the colorbar indicate the average value of the diagonal elements representing loci self-interactions.

Chromosome segregation was also investigated using chromosome contact maps of the same replicating chromosome systems. Contact maps were calculated at 250 bp resolution using the configurations from 3*T*_*f*_/4 ≤ *t* ≤ *T*_*f*_ (*i.e.*, when the replication state is constant) averaged over the five replicates for each case. We will denote the true contact maps for cases iii (Figure 8A) and vi (Figure 8B) as A and B and the sequence-equivalent maps as $\tilde{A}$ and $\tilde{B}$ , respectively. For both cases we can observe inter-daughter interactions indicated by the increased contact frequency within the off-diagonal regions of the true maps. The inter-daughter contacts are enriched in the case iii, where the system lacks topoisomerase, particlularly between the *Ter*s of *mll* (unreplicated region of *ml*) and *mrl* (unreplicated region of *mr*), which is consistent with our findings when using the degree of disentanglement (Figure 6C) and partitioning (Figure 7), and agree with experimental observations of topo-IV modulating daughter(/sister) cohesion (Lesterlin et al., 2012; Conin et al., 2022). Additionally, we can calculate sequence-equivalent maps to determine how these inter-daughter interactions would be represented in an experimental contact map generated from a 3C library of cells in this replication state, and under these topoisomerase conditions. The sequence equivalent maps, $\tilde{A}$ and $\tilde{B}$ , retain the characteristic primary diagonal and peaks at opposite corners indicative of circular chromosomes (inset Figure 8C). The effect of inter-daughter interactions are analyzed by comparing the average rates of loci self-interactions between the true and sequence-equivalent maps. The average loci self-interactions in true maps A and B are

$$\frac{\sum_{i}A_{ii}}{N}=0.083\;\text{and}\;\frac{\sum_{i}B_{ii}}{N}=0.091\text{,}$$ (3.1)

respectively. The average loci self-interactions in sequence-equivalent maps $\tilde{A}$ and $\tilde{B}$ are

$$\frac{\sum_{i}{\tilde{A}}_{ii}}{\tilde{N}}=0.146\;\text{and}\;\frac{\sum_{i}{\tilde{B}}_{ii}}{\tilde{N}}=0.121\text{,}$$ (3.2)

respectively.

Confoundingly, while one might anticipate a higher rate of loci self-interactions in $\tilde{B}$ relative to $\tilde{A}$ given the loci self-interactions in the true maps, the opposite case is true due to contributions from the inter-daughter interactions (Figure 8C), which are the result of precatenanes in the replicated daughters (Wang X. et al., 2008; Cebrián et al., 2015). This simple example is illustrative of how experimental contact maps not only encode an ensemble of chromosomes with different configurational states (Junier et al., 2015; Sekelja et al., 2016), but also encode an ensemble extending across an extra set of dimensions corresponding to the space of replication states. Sequence-equivalent maps have the further benefit of allowing one to observe changes in chromosome organization as a system follows a trajectory in configurational and replication state space. Using the simulations of case vi (Figure 6C), contact maps were calculated for ten time intervals of equal length (Supplementary Figures S6, S7). We see that the approach to a plateau in the degree of disentanglement (Figure 6C), which indicates the system is approaching a decatenaned state, is reflected in reduced differences in the sequence-equivalent contact maps (Supplementary Figures S6, S7).

### 3.3 Martini model

**FIGURE 9:**
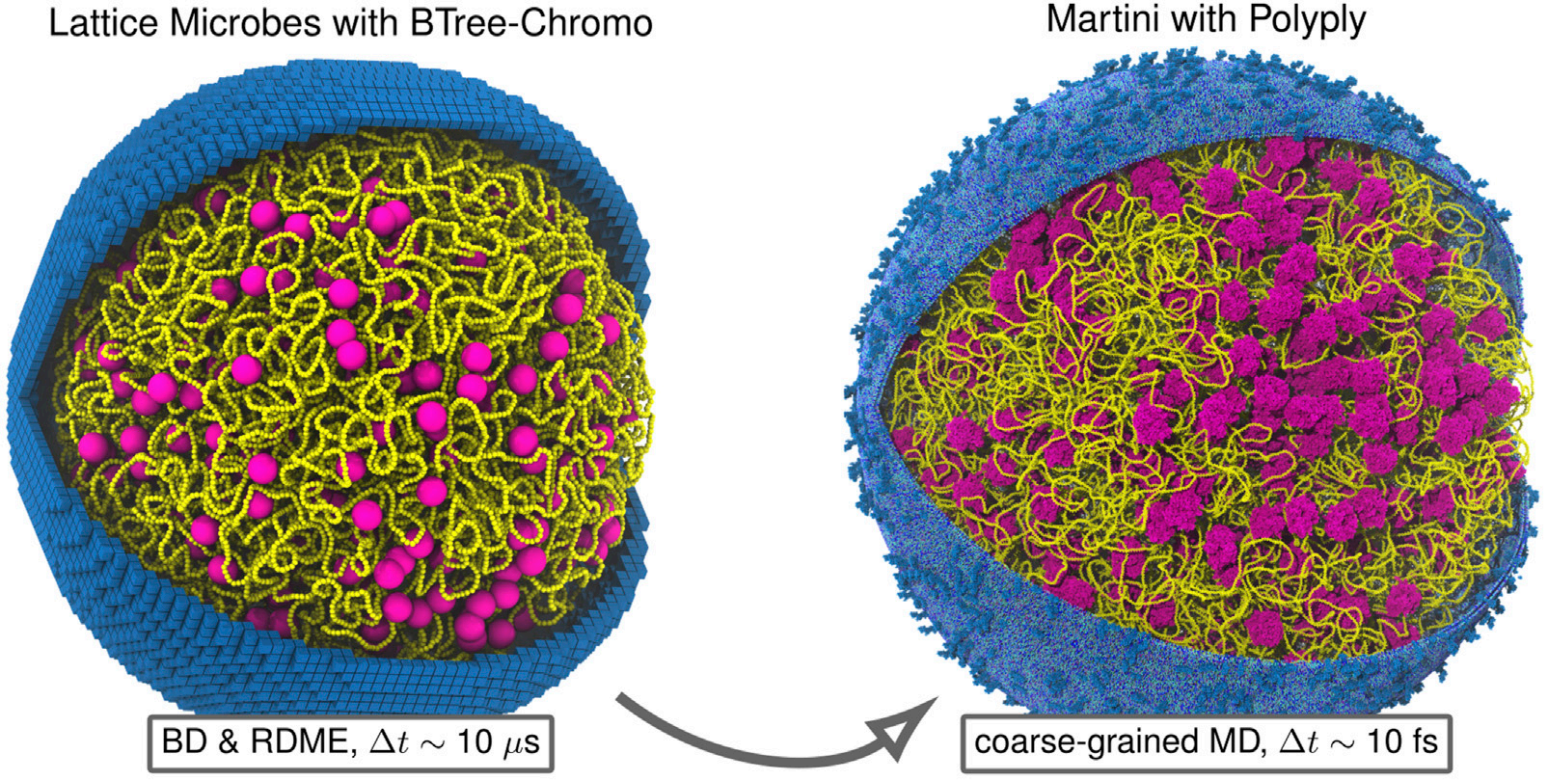
Backmapping Martini model of entire Syn3A cell: Example backmapping of polymer model of 200 nm radius Syn3A cell with a single unreplicated chromosome to near-atomistic resolution Martini representation using Polyply. For both representations we show the chromosome (yellow), ribosomes (magenta), and membrane (blue). The membrane in the Lattice Microbes representation is shown using the 8 nm cubic subvolumes used for reaction-diffusion master equation (RDME) simulations and the membrane in the Martini representation, which includes the lipid composition and membrane proteins of Syn3A (Thornburg et al., 2022), was generated using TS2CG (Pezeshkian et al., 2020). The two representations are complementary in that the combined polymer-RDME model resolves cell-wide chemical transformations over timescales comparable to the cell-cycle by neglecting detailed physical interactions among particles, while the Martini model alternatively resolves these detailed physical interactions among macromolecules over shorter timescales.

Using our new backmapping protocol, a Martini model of the Syn3A′s chromosome is constructed (Figure 9). With the aim of performing a molecular dynamics (MD) simulation, both starting configuration and topology are generated based on the previously described polymer model and the genome’s sequence. The resulting Martini model contains around 7 million Martini beads, representing the 34 million atoms constituting the chromosome.

The chromosome model is energy minimized in vacuum using Gromacs-2023 (Abraham et al., 2023). However, running an MD simulation, additionally requires the solvation and charge neutralization of the model. This step dramatically increases the number of particles in the simulation to over 500 million Martini beads. At the current stage, Gromacs can not handle systems of this size, which restrains us from further exploring the dynamics of the system.

However, to illustrate our DNA backmapping protocol, we model and simulate the previously described toy chromosome system of approximately one-tenth the size of the Syn3A. Before applying our chromosome modeling protocol to this toy model, we first sample an artificial 50 kbp sequence with the same relative nucleobase frequency as the Syn3A genome. The resulting Martini model is solvated in a 185 nm cubic box, neutralized, and subsequently, a physiological salt concentration of 0.15 M NaCl is added to the system. To incorporate the confinement effect of the membrane on the chromosome, an additional spherical boundary potential with a radius of 90 nm is added to the model. Note that in the Martini version of the toy system, we omitted to model the ribosomes.

**FIGURE 10:**
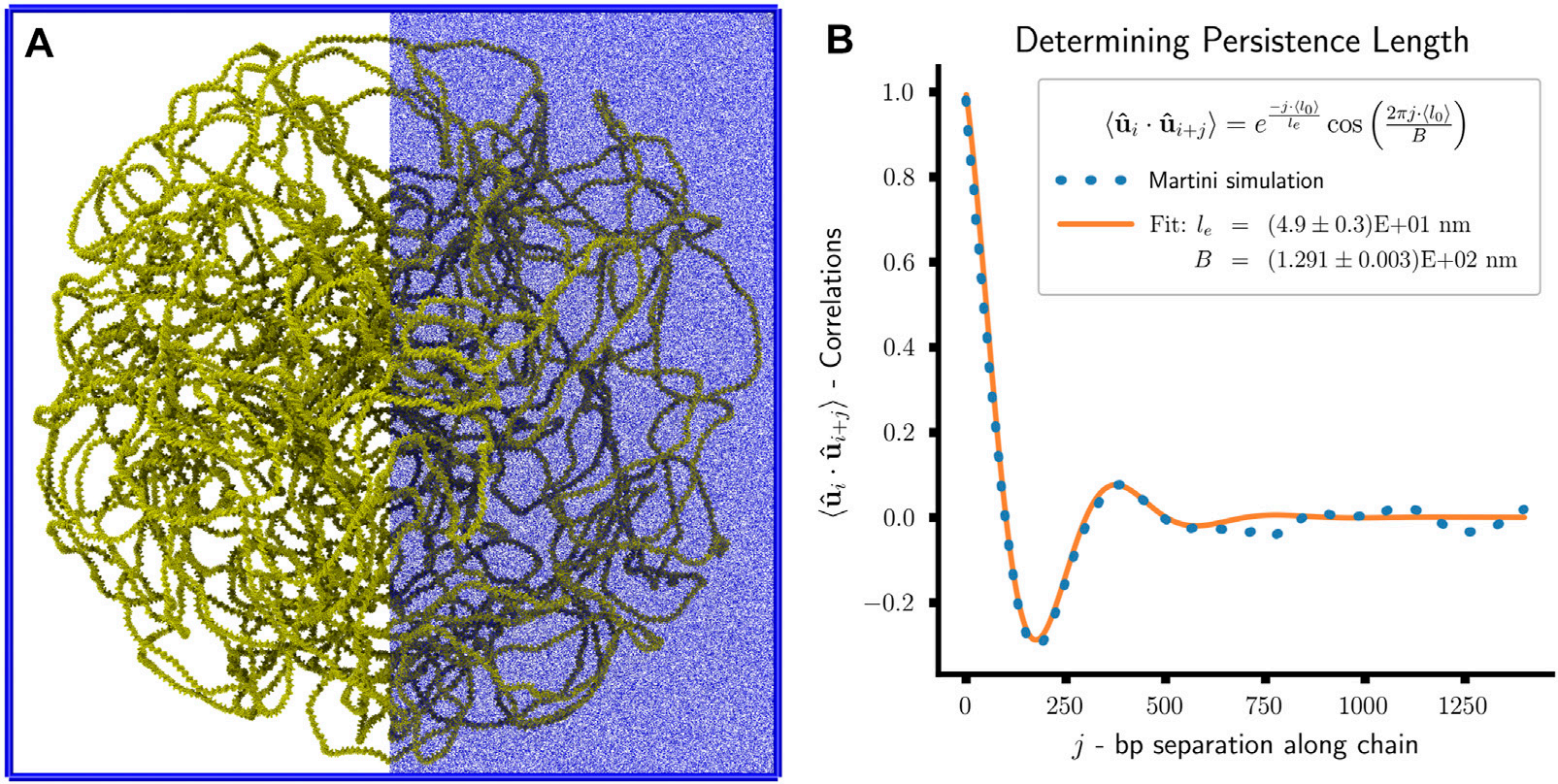
Martini simulation of toy system: **(A)** Snapshot of Martini simulation of toy system. The system consists of approximately 50 million Martini beads—chromosome 650,000 (yellow), water 50,528,240 (not shown), chloride ions 571,949 (blue), and sodium ions 671,949 (blue). The ions are only displayed on the right-half to enhance visual clarity. **(B)** Plot of bond vector correlations as a function of bp separation along the polymer chain and the least-squares fit of the effective persistence length, *l*_*e*_, and confinement length scale, B.

The final simulation consists of approximately 50 million Martini beads, representing over 500 million atoms (Figure 10A). First, we energy minimize and equilibrate the system before starting the production simulation, which is stable at a 20 fs timestep. In total, the system is simulated for 50 ns We note that on this short timescale, the chromosome will not fully equilibrate. Nevertheless, we have the ability to confirm that our backmapped model is consistent with the intended structure and observed sub-diffusive motion (*α* ≈ 0.87) of 10 bp segments of the Martini dsDNA (Supplementary Figure S5) that is consistent with the Brownian dynamics simulations of the full chromosome in the absence of ribosomes (Supplementary Figure S4).

A direct comparison between the polymer and Martini simulations is possible by analyzing the models’ persistence lengths, *l*_*p*_. For the Martini simulation, we determine the persistence length of the chromosomal DNA by calculating the orientational correlation of the bond vectors, ${\hat{u}}_{i}$ , connecting the centers of consecutive bps. In an idealized worm-like chain approximation, we expect the bond vectors to decorrelate exponentially along the chain, $\left\langle {\hat{u}}_{i}\cdot {\hat{u}}_{i+j}\right\rangle =e^{-j\left\langle l_{0}\right\rangle /l_{p}}$ , where $\left\langle l_{0}\right\rangle$ is the mean distance between bps.

However, calculating the bond vector correlations for the last 25 ns of the Martini simulation (Figure 10B) reveal a clear deviation from this idealized model. An additional oscillatory contribution is observed in the decay of the bond vector correlations, which can be attributed to the geometric confinement of the chromosome by the cell wall (Liu and Chakraborty, 2008; Cifra and Bleha, 2010; Castro-Villarreal and Ramírez, 2021). The resulting decay trend is well-captured by

$$\left\langle {\hat{u}}_{i}\cdot {\hat{u}}_{i+j}\right\rangle =e^{\frac{-j\left\langle l_{0}\right\rangle }{l_{e}}}\cdot \cos\left(\frac{2\pi j\left\langle l_{0}\right\rangle }{B}\right)\text{,}$$ (3.3)

where *l*_*e*_ is the effective persistence length of the DNA, and *B* is a length scale related to the confinement size (Liu and Chakraborty, 2008). By performing a least-squares fit of the model to our simulation, we find *l*_*e*_ = (4.9 ± 0.3)E+01 nm and *B* = (1.291 ± 0.003)E+02 nm. Considering the 45 nm persistence length of the polymer model, which is a chosen model parameter, we observe a qualitative agreement between the two models. The quantitative deviation can be attributed to the confinement reducing the chromosome’s conformational space and increasing its effective rigidity. In general, the measured *l*_*e*_ will be greater than or equal to *l*_*p*_ under confinement. However, the small amplitude of the fluctuations in the measured bond vector correlations indicates a moderate confinement regime, suggesting that *l*_*e*_ and *l*_*p*_ are comparable (Liu and Chakraborty, 2008).

## 4 Discussion

### 4.1 Study overview and methods

We developed a computational framework to investigate the minimal required components for chromosome replication and segregation in a genetically minimal bacterium, Syn3A. This framework is built around six major components: 1) a method to fold chromosomes around ribosome distributions originating from cryo-ET or other experimental measurements (Supplementary Figure S1), 2) an implementation of a 10 bp per monomer polymer model of dsDNA that includes its intrinsic mechanical properties (bending and twisting stiffness) and can be simulated using Brownian dynamics (Figures 1A–C), 3) algorithms that emulate the effect of known essential proteins that manipulate the chromosome — DNA-looping SMC complexes and strand-crossing type-II topoisomerases (Figure 1D; Supplementary Algorithms S1, S2), 4) a binary tree model to systematically describe nontrivial replication states and create accompanying 3D physical structures obeying the polymer model (Figure 2), 5) *in silico* chromosome contact maps of replicating chromosomes that capture intra- and inter-daughter interactions (Figure 3), and 6) a procedure mapping the chromosome to equivalent higher-resolution Martini whole-cell models using Polyply (Figure 9).

### 4.2 Key findings

Using the binary tree model of replication states, we have created a means to systematically describe nontrivial replication states that are known to be present in bacteria (Cooper and Helmstetter, 1968; Bremer and Dennis, 2008; Youngren et al., 2014). Previous simulations of replicating chromosomes have used either a set of fixed replication states (Wasim et al., 2021; 2023; Mitra et al., 2022b) or a pre-defined replication protocol (Mitra et al., 2022a). Our software implementation of this model enables users to create physical models of these states with the bond topology of nested theta structures (Figure 2A) and modify the states using computational equivalents of biological processes (Figure 2B). Furthermore, the aspects of the program used to create, manipulate (replicate asymmetrically at specific forks, replicate under well-stirred assumption), query (export bond topology, loci for true and sequence-equivalent maps, counts of genomic regions, etc.), and save replication states may be used independently from simulations of a physical model, which allows other researchers to use the program as a tool.

By combining the binary tree model with the Brownian dynamics model of the chromosomal dsDNA, we have developed a method to generate physics-based models of replicating chromosomes at 10 bp resolution, and simulate their time-evolution while undergoing diffusive motion and non-equilibrium replication events. Cryo-ET of Syn3A demonstrated that the ribosome distribution is near-uniform and the cytoplasm appears denser than other bacteria (Gilbert et al., 2021) and the chromosome itself, through excluded volume interactions with other macromolecular complexes (Dersch et al., 2022) and spatially localized transcription (Llopis et al., 2010), potentially represents the greatest influence on spatially heterogeneous reaction-diffusion processes within simulations of Syn3A (Thornburg et al., 2022).

After folding chromosomes organized as a fractal globule around ribosomes positions from cryo-ET (Gilbert et al., 2021), we measured the diffusion of complete 70S bacterial ribosomes. We find that configurations of the chromosomes create polymer meshworks that have voids containing ribosomes. Within these voids the ribosomes undergo nearly Brownian motion with diffusion constants lower than those observed in *E. coli* (Bakshi et al., 2012). We find that non-specific DNA-looping in the absence of a parAB*S* system compacts the chromosome, with the assumed number of loops based on proteomics of SMC-scpAB components (Table 1) reducing the radius of gyration of 100-monomer segments by approximately 35% (Figure 1E). Although this compaction is substantial, the chromosome can be still be replicated using our implementation of the train-track model without issues.

In the context of our model, we find that both DNA-looping and strand-crossings are necessary for the segregation of daughter chromosomes during and after replication, which is in agreement with Syn3A′s gene essentiality data for SMC-complexes and type-II topoisomerases from transposon mutagenesis experiments (Breuer et al., 2019). We analyzed the time-course of chromosome segregation in a toy system by dividing it into two processes, disentanglement of the daughter chromosomes (Figure 6) and partitioning of the daughter chromosomes into distinct volumes (Figure 7). The system cannot be disentangled when no loops are present. Increasing the number of loops leads to disentanglement of the first generation of daughters, but that process will stall if topoisomerase is absent and the topological restraints cannot be resolved (Figure 6C), which is in agreement with experiments (Wang X. et al., 2008). Additionally, if there are loops and no topoisomerase, subsequent generations will be even more entangled due to replication occurring in the daughters already compacted by loops (Figure 6C). This coordinated role between SMC complexes and topo-IV has been observed in *E. coli* (Zawadzki et al., 2015; Nolivos et al., 2016; Mäkelä and Sherratt, 2020). Identical behavior is observed in the partitioning of the daughters (Figure 7), but the partitioning occurs over a slower timescale than the disentanglement, with the partitioning less than 50% complete on average in case vi, where the daughters are almost completely disentangled. Based on this, successful disentanglement is necessary in our model for partitioning to proceed. It is qualitatively clear that partitioning lags behind disentanglement in the proof-of-concept simulation of the full chromosome undergoing simultaneous replication and segregation (Supplementary Video SV2), but we are encouraged by the preliminary result for the degree of disentanglement demonstrating that SMC complexes and topo-IV are sufficient at the chromosome-scale (Supplementary Figure S8).

Overall, these findings regarding the influence of SMC complexes and topoisomerases on chromosome segregation are consistent with computational studies of eukaryotic sister chromatids (Goloborodko et al., 2016a) and show that the same mechanisms are capable of segregating nested theta structures in bacteria. While we model the chromosome as a homopolymer rather than a heteropolymer, the energy landscape picture of proteins within a funnel (Bryngelson et al., 1995; Onuchic et al., 1997) is relevant when interpreting the process of chromosome segregation. The ATP-consuming process of loop-extrusion isolates knots and causes the system to approach energetic barriers representing these topological restraints within the system. Our model’s periodic action of topoisomerases then lowers the barriers and loop-extrusion drives the system over the lowered barriers. We found that neither of these effects is sufficient in isolation, and the combination of ATP-consuming driving forces and lowered barriers enable the departure from a local energy minimum with a more-knotted topology into a new energy minimum with a less-knotted topology, which is consistent with previous computational studies on knotted chromosome topologies (Racko et al., 2018; Orlandini et al., 2019). These processes are akin to the role of protein-folding chaperones in resolving kinetically trapped misfolded proteins in a rugged energy landscape (Todd et al., 1996; Thirumalai et al., 2019).

Previous studies have calculated *in silico* chromosome contact maps of replicating bacterial chromosomes (Wasim et al., 2021; 2023), but to the best of our knowledge, did not include inter-daughter contacts. Using our model, we have created a procedure to calculate true maps that include inter-daughter contacts and convert those maps extending over the full DNA content of the replicating chromosome system back to the sequence-equivalent maps that would be measured by experimental 3C methods (Figure 3). This not only elucidates variations in the sequence-equivalent maps due to differing spatial organization of chromosomes in identical replication states (Figure 8), but also enables the comparison of maps originating from chromosomes in different replication states and the creation of maps representing a mixture of replication states. Features in contact maps that are attributed to processes during replication and chromosome segregation have been previously reported in synchronized *Caulobacter crescentus* cells (Le et al., 2013) and *E. coli* topo-IV knockout studies (Conin et al., 2022).

Using Polyply, we showed that we can obtain a starting structure of the entire Syn3A chromosome at near-atomic resolution, ready for subsequent sampling of its configuration space using molecular dynamics. Previous dynamics simulations of entire chromosomes are either based on simplified (1-2 bead per bp) models or are restricted to simulating smaller, viral genomes and nanostructures (Maffeo and Aksimentiev, 2020; Sengar et al., 2021).

### 4.3 Limitations

There is no sequence-specificity in the homopolymer model of replicating chromosomes beyond specific landmark monomers such as *Ori*s and *Ter*s, and there is no means to represent ssDNA. This limitation precludes us from modeling the unique molecular structures of the bubble during replication initation (Shimizu et al., 2016) and replisome during replication (Maffeo et al., 2022). The essentiality of HU in Syn3A despite its reduced proteomics count and high-affinity for structurally deformed DNA (Kamashev, 2000) suggests a role in DNA replication, which is further supported by the *Ori*:*Ter* ratio of *B. subtilis* being reduced upon HU deletion (Karaboja and Wang, 2022). However, in contrast to *E. coli* where HU/IHF has a well-defined role of stabilizing bent dsDNA in DnaA-based replication at an *oriC* (Yoshida et al., 2023), there is a lack of clarity regarding HU’s role in Syn3A′s more minimalistic *oriC* (Richardson et al., 2019; Thornburg et al., 2019). In a similar vein, although the binary tree model fully describes topologies of nontrivial replication states that may be undergoing asymmetric replication, the absence of ssDNA prevents us from making the distinction between leading and lagging strands, which would be at the extreme end in opposite directions (clockwise vs. counter-clockwise) on the left and right daughters.

In the chromosome-scale polymer model, we neglected hydrodynamic interactions and did not directly include electrostatics beyond the parameterization of the persistence length, we feel the ability to backmap the system to a Martini representation with near-atomistic detail helps resolve this deficiency by providing information about the effect of neglecting those interactions. In particular, to address the viscoelastic nature of the medium, which was neglected in the Brownian dynamics model, one could simulate the polymer model using dissipative particle dynamics (DPD) (Español and Warren, 2017), where the memory function encoding non-Markovian dynamics due to the medium is constructed (Klippenstein et al., 2021) from whole-cell Martini simulations (Stevens et al., 2023). The Brownian dynamics timesteps (Δ*t* = 0.1 ns) are much smaller than the timescales of loop-extrusion events ( ∼1 s) (Ryu et al., 2021), and vastly smaller than Syn3A′s cell-cycle ( ∼6600 s) (Breuer et al., 2019; Thornburg et al., 2022). To circumvent this, we used energy minimizations to relax the chromosome after non-equilibrium loop-extrusion steps, which helped to accelerate the simulations. However, this came at the cost of disconnecting the Brownian dynamics simulation time from the biological time of the loop-extrusion events. The current implementation of the code calls LAMMPS (Thompson et al., 2022) to run the Brownian dynamic simulations using multiple CPU-threads with OpenMP. Although this approach was sufficiently fast, in the course of the study it has become clear that moving the simulations to the GPU would offer a significant improvement.

### 4.4 Future directions

Now that we have created a computational model of Syn3A′s chromosome that includes replication and segregation of nontrivial replication states, we intend to integrate it with the 4D-WCM of Syn3A (Thornburg et al., 2022) to extend its predictive capabilities to the full cell-cycle. Information concerning the spatial coordinates of the replicating genome will be sent to the 4D-WCM and information regarding reaction events will be returned, similar approaches have been used by other researchers (Popov et al., 2016). Two immediate applications are the modeling of DnaA filamentation leading to formation of the replication bubble and the dynamic formation of polysomes based on translational activity. Given the absence of regulatory elements in Syn3A, an open-question is if the arrangement of genes can serve as a means of regulation (Chatterjee et al., 2021; Geng et al., 2022) as a result of the mechanochemical coupling between transcription and supercoiling (Chong et al., 2014; Kim et al., 2019). Following transcription events in the 4D-WCM, dynamically applying torsional strain to the chromosome model would enable local configurational changes in genes, thereby modulating their transcriptional propensity.

While the methods described in this study enable us to calculate *in silico* chromosome contact maps whose *Ori*:*Ter* ratio matches experimental qPCR measurements of 3.4 (Thornburg et al., 2022) by using a mixture of replication states with different ratios, there is a lack of clarity about relative weights of these states. Furthermore, there are a vast multitude of compatible replication microstates for each *Ori*:*Ter* ratio. Given that we now have a means to generate sequence-equivalent *in silico* contact maps of chromosomes in different replication states, this motivates the development of a protocol to deconvolve experimental maps generated from populations of unsynchronized cells (Junier et al., 2015; Sefer et al., 2016; Carstens et al., 2020; Zhou et al., 2021; Rowland et al., 2022) to determine the subpopulations of cells in different replication states. The respective replication states would then be found by an inversion of subpopulation contact maps (Supplementary Figures S6, S7) from their sequence-equivalent form to the true contact maps (Figure 3). We note that this proposed methodology faces two challenges: 1) the solution requires knowledge of sequence-equivalent contact maps for replication microstates and 2) even with that information, the problem likely remains underdetermined if only subject to the example set of constraints (Section 2.5) and not more informative constraints such as DNA abundance distributions (Bhat et al., 2022). Assuming further performance improvements of the simulation software, this study helps to address the first issue, but the second issue will need to be resolved.

All simulations of chromosome segregation in this study used a spherical confinement reflecting the observed morphology of SynX-series (Gilbert et al., 2021; Pelletier et al., 2021) and *M. mycoides* (Rideau et al., 2022) cells. Varying confinement over the cell-cycle will allow us to test entropic segregation in shapes with long aspect-ratios (Jun and Mulder, 2006; Jung and Ha, 2010; Jung et al., 2012; Youngren et al., 2014).

Simulations with the Martini model are limited in the description of DNA strand hybridization. To keep the strands together, an elastic network is used. Ongoing efforts are directed to include additional (virtual) bead types that provide a more accurate description of the directed hydrogen bonds that give rise to specific base pairing. Another challenge is to capture the replicating chromosome when creating whole-cell Martini models of different stages of the cell cycle. To this end, a Martini model of a complete replisome has to be constructed and integrated into our chromosome modeling protocol. As part of our DNA backmapping algorithm, we plan to support the incorporation of protein-DNA complexes, thereby facilitating the construction of complete replication forks.

## Data Availability

All software used for simulations and analysis in this study is open-source and listed in Supplementary Table S1. Software used for visualization is publicly available and listed in Supplementary Table S1.
