# Supplementary material for: Dynamics of chromosome organization in a minimal bacterial cell
Source: Front Cell Dev Biol. 2023 Aug 9;11:1214962. doi: 10.3389/fcell.2023.1214962 (PMC10445541; doi:10.3389/fcell.2023.1214962)
Supplement: Supplementary file 1 [file DataSheet1.pdf]

Supplementary Information: Dynamics of Chromosome Organization in a Minimal Bacterial Cell

Benjamin R. Gilbert [1], **Zane R. Thornburg** [1], **Troy A. Brier** [1], **Jan A. Stevens** [2], **Fabian Grünewald** [2], **John E. Stone** [3,4], **Siewert J. Marrink** [2], **and Zaida Luthey-Schulten** [1,4,5]

[1]Department of Chemistry, University of Illinois at Urbana-Champaign, Urbana, IL 61801, USA

[2]Molecular Dynamics Group, Groningen Biomolecular Sciences and Biotechnology Institute, University of Groningen, Groningen, Netherlands

[3]NVIDIA Corporation

[4]NIH Center for Macromolecular Modeling and Bioinformatics, Beckman Institute, University of Illinois at Urbana-Champaign, Urbana, IL 61801, USA
[5]NSF Center for the Physics of Living Cells, Department of Physics, University of Illinoist at Urbana-Champaign, Urbana, IL 61801, USA

Supplementary Analyses

Spherical Monomers. An empirical formula (Tirado et al., 1984) for the macroscopic translational damping of a cylinder with of length L and diameter d is given by

$$\gamma^{\mathrm{T}}_{\mathrm{cyl.}} = \frac{3\pi\eta L}{\log p + v}, \tag{S1}$$

where $p = L/d$ and the end-effect correction v is

$$v = 0.3212 + 0.565p^{-1} - 0.100p^{-2}. \tag{S2}$$

Comparison to experiments on short oligonucleotides show that this formula is valid down to $p = 1.4$ (Eimer and Pecora, 1991). If we were to model 10 bp segments as cylinders, then $L = 3.4$ nm, $d = 2.0$ nm, and $p = 1.7$. The discrepancy in approximating the cylinders using the Stokes-Einstein equation for spherical particles with hydrodynamic radius $r = L/2$ is then

$$\frac{\gamma^{\mathrm{T}}}{\gamma^{\mathrm{T}}_{\mathrm{cyl.}}} = \log p + v \approx 1.15, \tag{S3}$$

which we consider to be an acceptable approximation for our chromosome-scale modeling.

Diffusion. Given a set of N particle coordinates at each trajectory frame of a replicate simulation, $\{\boldsymbol{x}_i(t)\}$, the mean-squared displacement (MSD) between times t and $t+\tau$ is calculated as

$$\mathrm{MSD}(\tau) = \left\langle |\boldsymbol{x}(t+\tau) - \boldsymbol{x}(t)|^2 \right\rangle = \frac{1}{N}\sum_i |\boldsymbol{x}_i(t+\tau) - \boldsymbol{x}_i(t)|^2. \tag{S4}$$

Given a set of MSD values for trajectory frames of a replicate simulation, $\{\mathrm{MSD}(\tau_i)\}$, the Brownian diffusion constant (Oliveira et al., 2019; Muñoz-Gil et al., 2021) for 3D diffusion is calculated as the least-squares solution to

$$\mathrm{MSD}(\tau_i) = 6D\tau_i. \tag{S5}$$

Given a set of MSD values for trajectory frames of a replicate simulation, $\{\mathrm{MSD}(\tau_i)\}$, the anomalous diffusion law is

$$\mathrm{MSD}(\tau_i) = D_a \tau_i^{\alpha}, \tag{S6}$$

where D_a is the anomalous diffusion constant and α is the power-law exponent (Oliveira et al., 2019; Muñoz-Gil et al., 2021). These are then calculated as the least-squares solution to

$$\log\left(\frac{\mathrm{MSD}(\tau_i)}{\mathrm{MSD}(\tau_1)}\right) = \alpha \log\left(\frac{\tau_i}{\tau_1}\right) + \log(D_a/D_a). \tag{S7}$$

Radial Distribution Functions. Due to the absence of a clear nucleoid region in cryo-ET of Syn3A (Gilbert et al., 2021), radial distribution functions were used to characterize the crowding effects of DNA on the ribosomes distributed near-uniformly throughout the cytoplasm. Smooth estimates of radial distribution functions were calculated using a spectral Monte Carlo method (Patrone and Rosch, 2017). Consider that the radial distribution function is expanded using a set of smooth orthogonal basis functions, $\phi_j(r)$,

$$g(r) \approx g_M(r) = \sum_{j=0}^{M} a_j \phi_j(r), \tag{S8}$$

where these functions are orthogonal on the interval, $[0, r_c]$, extending from the test particle at the origin to a cutoff radius, r_c, with respect to the weight function, $w(r)$,

$$\int_0^{r_c} \mathrm{d}r w(r) \phi_j(r) \phi_k(r) = W_{jk} \delta_{jk}. \tag{S9}$$

We can use the orthogonality relation to solve for the spectral coefficients

$$\begin{aligned} \int_0^{r_c} \mathrm{d}r w(r) \phi_k(r) g(r) &= \int_0^{r_c} \mathrm{d}r w(r) \phi_k(r) \left[\sum_{j=0}^{\infty} a_j \phi_j(r) \right] \\ &= \sum_{j=0}^{\infty} a_j \int_0^{r_c} \mathrm{d}r w(r) \phi_k(r) \phi_j(r) \\ &= \sum_{j=0}^{\infty} a_j W_{jk} \delta_{jk} \\ &= a_k W_{kk} \end{aligned} \tag{S10}$$

which leads to

$$\begin{aligned} a_k &= \frac{1}{W_{kk}} \int_0^{r_c} \mathrm{d}r w(r) \phi_k(r) g(r) \\ &= \frac{1}{W_{kk}} \int_0^{r_c} \mathrm{d}r w(r) \phi_k(r) \frac{N(r)}{4\pi r^2 \rho}. \end{aligned} \tag{S11}$$

The quantity $N(r)\mathrm{d}r$ is the expected number of particles in a spherical shell of radius r and thickness $\mathrm{d}r$ centered about a particle at the origin. The probability density, $\mathsf{n}(r)$, of finding a single particle in such a shell is proportional to this

$$\mathsf{n}(r)\mathrm{d}r \propto N(r)\mathrm{d}r \quad \rightarrow \quad \mathsf{n}(r)\mathrm{d}r = \left(\frac{N(r)}{\mathsf{N}(r_c)} \right) \mathrm{d}r, \text{ where } \mathsf{N}(r_c) = \int_0^{r_c} \mathrm{d}r N(r) \tag{S12}$$

and we may rewrite the equation for the spectral coefficients in terms of an expectation value over the probability density $\mathsf{n}(r)$

$$\begin{aligned} a_k &= \frac{1}{W_{kk}} \times \frac{\mathsf{N}(r_c)}{4\pi\rho} \times \int_0^{r_c} \mathrm{d}r \left(\frac{w(r)\phi_k(r)}{r^2} \right) \frac{N(r)}{\mathsf{N}(r_c)} \\ &= \frac{1}{W_{kk}} \times \frac{\mathsf{N}(r_c)}{4\pi\rho} \times \int_0^{r_c} \mathrm{d}r \left(\frac{w(r)\phi_k(r)}{r^2} \right) \mathsf{n}(r) \\ &= \frac{1}{W_{kk}} \times \frac{\mathsf{N}(r_c)}{4\pi\rho} \times \left\langle \frac{w(r)\phi_k(r)}{r^2} \right\rangle_{\mathsf{n}(r)} \end{aligned} \tag{S13}$$

This expectation value can be estimated using Monte Carlo integration of pairwise distances that are sampled from the Brownian dynamics simulations

$$\left\langle \frac{w(r)\phi_k(r)}{r^2} \right\rangle_{\mathsf{n}(r)} \approx \frac{1}{N_{\text{pairs}}} \sum_{j=1}^{N_{\text{pairs}}} \frac{w(r_j)\phi_k(r_j)}{r_j^2} \tag{S14}$$

In the case of the radial distribution function of DNA monomers about a ribosome at the origin, the number of pairs in a single trajectory frame is given by a sum of the the number of ribosomes, N_{ribo}, where for j-th ribosome we then sum over the DNA monomers within a distance r_c, denoted as $N_{\text{DNA}}(j, r_c)$,

$$\frac{1}{N_{\text{pairs}}} \sum_{j=1}^{N_{\text{pairs}}} \frac{w(r_j)\phi_k(r_j)}{r_j^2} = \frac{1}{N_{\text{ribo}}} \sum_{j=1}^{N_{\text{ribo}}} \left(\frac{1}{N_{\text{DNA}}(j, r_c)} \sum_{i=1}^{N_{\text{DNA}}} \left[\frac{w(r_{ji})\phi_k(r_{ji})}{r_{ji}^2} \times \Theta(r_c - r_{ji}) \right] \right) \tag{S15}$$

and

$$N_{\mathsf{DNA}}(j,r_c) = \sum_{i=1}^{N_{\mathsf{DNA}}} \Theta(r_c - r_{ji}), \tag{S16}$$

where

$$r_{ji} = |\boldsymbol{x}_j^{\mathsf{ribo}} - \boldsymbol{x}_i^{\mathsf{DNA}}|. \tag{S17}$$

For notational convenience, we will continue denoting this as a sum over the pairs. Using the change of variable

$$\zeta(r) = \frac{(2r - r_c)}{r_c}, \quad r(\zeta) = \frac{r_c(\zeta+1)}{2}, \quad \text{and} \quad \mathrm{d}\zeta = \frac{2}{r_c}\mathrm{d}r \tag{S18}$$

we can define Chebyshev polynomials of the first kind, $T_n(x)$, which are given by the recurrence relation

$$\begin{aligned} &T_0(x) = 1 \\ &T_1(x) = x \\ &T_n(x) = 2xT_{n-1}(x) - T_{n-2}(x), \quad \text{where} \quad n \geq 2 \end{aligned} \tag{S19}$$

and follow the orthogonality relation

$$\int_{-1}^{1} \mathrm{d}x \frac{T_n(x)T_m(x)}{\sqrt{1-x^2}} = W_{nm}\delta_{nm}, \quad \text{where} \quad W_{nm} = \begin{cases} 0 & n \neq m \\ \pi & n = m = 0 \\ \pi/2 & n = m \neq 0 \end{cases} \tag{S20}$$

on the interval $[0, r_c]$ (Press et al., 2007). We then use these Chebyshev polynomials in the transformed variable, ζ, as our basis functions and their accompanying weight function

$$w(r) = \omega\big(\zeta(r)\big), \quad \phi_k(r) = T_k\big(\zeta(r)\big), \quad \text{and} \quad \mathsf{n}(r) = \tilde{\mathsf{n}}\big(\zeta(r)\big). \tag{S21}$$

This implicitly changes the expectation value as follows

$$\begin{aligned} \left\langle \frac{w(r)\phi_k(r)}{r^2} \right\rangle_{\mathsf{n}(r)} &= \int_0^{r_c} \mathrm{d}r \left(\frac{w(r)\phi_k(r)}{r^2} \right) \mathsf{n}(r) \\ &= \frac{2}{r_c} \int_{-1}^{1} \mathrm{d}\zeta \left(\frac{\omega(\zeta)T_k(\zeta)}{r(\zeta)^2} \right) \tilde{\mathsf{n}}(\zeta) \\ &= \frac{8}{r_c^3} \int_{-1}^{1} \mathrm{d}\zeta \left(\frac{\omega(\zeta)T_k(\zeta)}{(\zeta+1)^2} \right) \tilde{\mathsf{n}}(\zeta) \\ &= \frac{8}{r_c^3} \left\langle \frac{\omega(\zeta)T_k(\zeta)}{(\zeta+1)^2} \right\rangle_{\tilde{\mathsf{n}}(\zeta)}, \end{aligned} \tag{S22}$$

where this is now an expectation value over the probability density of separation distances, $\tilde{\mathsf{n}}(\zeta)$, in the transformed variable, ζ, which is defined on the interval $[-1, 1]$. Defining $\zeta_j = \zeta(r_j)$, we can now estimate this expectation value again as a sum over the same pairs

$$\left\langle \frac{\omega(\zeta)T_k(\zeta)}{(\zeta+1)^2} \right\rangle_{\tilde{\mathsf{n}}(\zeta)} \approx \frac{1}{N_{\mathsf{pairs}}} \sum_{i=1}^{N_{\mathsf{pairs}}} \frac{\omega(\zeta_j)T_k(\zeta_j)}{(\zeta_j+1)^2} \tag{S23}$$

The spectral coefficients for an expansion of the ribosome-DNA radial distribution function in terms of Chebyshev polynomials are estimated as

$$\begin{aligned} a_k &\approx \frac{1}{W_{kk}} \times \frac{\mathsf{N}_{\mathsf{DNA}}(r_c)}{4\pi\rho_{\mathsf{DNA}}} \times \frac{8}{r_c^3} \times \left[\frac{1}{N_{\mathsf{pairs}}} \sum_{j=1}^{N_{\mathsf{pairs}}} \frac{\omega(\zeta_j)T_k(\zeta_j)}{(\zeta_j+1)^2} \right] \\ &= \frac{8}{3W_{kk}} \times \frac{\left[\mathsf{N}_{\mathsf{DNA}}(r_c)/V_{\mathsf{sphere}}(r_c)\right]}{\rho_{\mathsf{DNA}}} \times \left[\frac{1}{N_{\mathsf{pairs}}} \sum_{j=1}^{N_{\mathsf{pairs}}} \frac{\omega(\zeta_j)T_k(\zeta_j)}{(\zeta_j+1)^2} \right] \end{aligned} \tag{S24}$$

and $\mathsf{N}_{\mathsf{DNA}}(r_c)$ can estimated as

$$\mathsf{N}_{\mathsf{DNA}}(r_c) \approx \frac{1}{N_{\mathsf{ribo}}} \sum_{j=1}^{N_{\mathsf{ribo}}} N_{\mathsf{DNA}}(j, r_c). \tag{S25}$$

The first factor in the estimator for the coefficients is a normalization for the selected basis functions and the second factor is the ratio of the average local density of DNA within the cutoff radius, $\rho_{\text{DNA}}(r_c) = \text{N}_{\text{DNA}}(r_c)/V_{\text{sphere}}(r_c)$, relative to the bulk DNA density. After estimating the spectral coefficients, the radial distribution function can be estimated to an arbitrary resolution in spatial distance. We found that the first 100 modes were sufficient to estimate smooth radial distribution functions with a negligible oscillations at the boundaries (Figure 5C).

Ideal Partitioning. To determine the ideal partitioning of a sphere into two volumes, we exploit the spherical symmetry and consider the equation of a semi-circle in the x-y plane to calculate the relative volumes, V_l and V_r, using the method of disks

$$y = \sqrt{r^2 - x^2} \quad \textbf{(S26)}$$

leads to the total volume given by

$$V = \pi \int_{-r}^{r} \mathrm{d}x y^2 = \pi \int_{-r}^{r} \mathrm{d}x (r^2 - x^2) \quad \textbf{(S27)}$$

Using the change of variable

$$\xi = \frac{x}{r}, \quad \text{and} \quad \mathrm{d}\xi = \frac{\mathrm{d}x}{r} \quad \textbf{(S28)}$$

we rewrite this in terms of a unit-sphere, and then consider α as the fraction of the distance between poles of the sphere at $\xi = -1$ and $\xi = 1$ to solve for two volume terms for the respective daughters in terms of α

$$\begin{aligned} V &= \pi r^3 \int_{-1}^{1} \mathrm{d}\xi (1 - \xi^2) \\ &= \pi r^3 \left[\int_{-1}^{-1+2\alpha} \mathrm{d}\xi (1 - \xi^2) + \int_{-1+2\alpha}^{1} \mathrm{d}\xi (1 - \xi^2) \right] \\ &= \frac{4\pi r^3}{3} (3 - 2\alpha)\alpha^2 + \frac{4\pi r^3}{3} (\alpha - 1)^2 (2\alpha + 1) \\ &= V_T (3 - 2\alpha)\alpha^2 + V_T (\alpha - 1)^2 (2\alpha + 1) \\ &= V_l(\alpha) + V_r(\alpha), \end{aligned} \quad \textbf{(S29)}$$

where V_T is the total volume of the sphere. Now we must solve for α such that

$$\frac{N_l}{N_l + N_r} = \frac{V_l(\alpha)}{V_T} = (3 - 2\alpha)\alpha^2 \quad \textbf{(S30)}$$

or

$$\frac{N_r}{N_l + N_r} = \frac{V_r(\alpha)}{V_T} = (\alpha - 1)^2 (2\alpha + 1). \quad \textbf{(S31)}$$

Assuming a uniform density of monomers ρ, the mass of one of these volumes is $m_{(l/r)}(\alpha) = \rho V_{(l/r)}(\alpha)$, and moments of these about y/z-axis are given by

$$\begin{aligned} M_l(\alpha) &= \rho \pi r^4 \int_{-1}^{-1+2\alpha} \mathrm{d}\xi (1 - \xi^2)\xi \\ &= r \times \left[\rho \frac{4\pi r^3}{3} \right] \times \left(-3(\alpha - 1)^2 \alpha^2 \right) \\ &= r \times \rho V_T \times \left(-3(\alpha - 1)^2 \alpha^2 \right) \end{aligned} \quad \textbf{(S32)}$$

and

$$\begin{aligned} M_r(\alpha) &= \rho \pi r^4 \int_{-1+2\alpha}^{1} \mathrm{d}\xi (1 - \xi^2)\xi \\ &= r \times \left[\rho \frac{4\pi r^3}{3} \right] \times \left(3(\alpha - 1)^2 \alpha^2 \right) \\ &= r \times \rho V_T \times \left(3(\alpha - 1)^2 \alpha^2 \right). \end{aligned} \quad \textbf{(S33)}$$

The centroids of these two volumes are located at

$$\bar{x}_l(\alpha) = \frac{M_l(\alpha)}{m_l(\alpha)} = -3r \frac{(\alpha - 1)^2 \alpha^2}{(3 - 2\alpha)\alpha^2} = -3r \frac{(\alpha - 1)^2}{(3 - 2\alpha)} \quad \textbf{(S34)}$$

and

$$\bar{x}_r(\alpha) = \frac{M_r(\alpha)}{m_r(\alpha)} = 3r\frac{(\alpha-1)^2\alpha^2}{(\alpha-1)^2(2\alpha+1)} = 3r\frac{\alpha^2}{(2\alpha+1)}. \quad \textbf{(S35)}$$

The distance between the centers of mass of daughters with N_l and N_r monomers when ideally partitioned is then

$$\begin{aligned} L_{\text{partition}}(N_l, N_r, r) &= L_{\text{partition}}(\alpha, r) \\ &= \bar{x}_r(\alpha) - \bar{x}_l(\alpha) \\ &= 3r\left[\frac{\alpha^2}{(2\alpha+1)} + \frac{(\alpha-1)^2}{(3-2\alpha)}\right]. \end{aligned} \quad \textbf{(S36)}$$

***In silico* Contact Calculations.** In this section we will present how we solve the forward problem of generating chromosome contact maps from our mechanistic model of Syn3A's chromosome. A single unreplicated chromosome is comprised of N monomers and its configurational state is given by the set of N coordinate vectors $\{\boldsymbol{x}_i\}$. For the purposes of calculating chromosome contacts at bp per locus resolutions coarser than the monomer size (10 bp), the chromosome is coarse-grained by dividing it into M loci comprised of regions of n_j bonded monomers in a contiguous series. The configurational state of the j-th loci is given by the set of n_j coordinate vectors, $\{\boldsymbol{x}_i^j\}$. The pairwise distance between monomer i of locus j and monomer i' of locus j' is denoted $r_{i,i'}^{j,j'} = |\boldsymbol{x}_i^j - \boldsymbol{x}_{i'}^{j'}|$. Defining $Q_{j,j'}$ as the number of pairwise distances between loci j and j' there are two cases. Case I ($j \neq j'$) with $Q_{j,j'} = n_j \times n_{j'}$ distances

$$\begin{aligned} \mathsf{R}_{j,j'} = \{ & r_{1,1}^{j,j'}, r_{1,2}^{j,j'}, \ldots, r_{1,n_{j'}}^{j,j'}, \\ & r_{2,1}^{j,j'}, r_{2,2}^{j,j'}, \ldots, r_{2,n_{j'}}^{j,j'}, \\ & \vdots \\ & r_{n_j,1}^{j,j'}, r_{n_j,2}^{j,j'}, \ldots, r_{n_j,n_{j'}}^{j,j'} \} \end{aligned} \quad \textbf{(S37)}$$

and case II ($j = j'$) with $Q_{j,j'} = n_j \times (n_{j'} - 1)/2$ distances

$$\begin{aligned} \mathsf{R}_{j,j'} = \{ & r_{1,2}^{j,j'}, r_{1,3}^{j,j'}, \ldots, r_{1,n_{j'}}^{j,j'}, \\ & r_{2,3}^{j,j'}, r_{2,4}^{j,j'}, \ldots, r_{2,n_{j'}}^{j,j'}, \\ & \vdots \\ & r_{(n_j-1),n_{j'}}^{j,j'} \}. \end{aligned} \quad \textbf{(S38)}$$

For both cases, we may equivalently write the set of loci(j)-loci(j') distances using a single index ranging from 1 to $Q_{j,j'}$ as

$$\mathsf{R}_{j,j'} = \{r_1^{j,j'}, r_2^{j,j'}, \ldots, r_{(Q_{j,j'}-1)}^{j,j'}, r_{Q_{j,j'}}^{j,j'}\}. \quad \textbf{(S39)}$$

Although we use loci of uniform size in this study, it is possible to use non-uniform sizes, and at 10 bp per monomer the loci could be comprised of regions partitioned by restriction enzyme cut sites (Lieberman-Aiden et al., 2009; Crémazy et al., 2018).

A number of transfer functions connecting loci/particle/monomer distances $\mathsf{D}_{j,j'}$ to the relative contact frequency $\mathsf{F}_{j,j'}$ have been used in past studies (Meluzzi and Arya, 2012; Serra et al., 2015; Le Treut et al., 2018; Abbas et al., 2019; MacKay and Kusalik, 2020). However, in our case the loci(j)-loci(j') distances measured are the set of distances between their constituent monomers and we cannot use an iterative approach to refine a parameterization of the transfer function (Zhang et al., 2013). We instead quantify the contact probability through a simplified model of the crosslinking process (Hoffman et al., 2015). We make the following assumptions about the crosslinker: i) crosslinker (formaldehyde) has diffused uniformly throughout the cell, ii) proteins are distributed uniformly throughout the cytoplasm, iii) the crosslinker acts in a manner independent of the DNA sequence, iv) crosslinking occurs pairwise between monomers and is independent of other crosslinking events, v) crosslinking probability $p_{cl}(r)$ is a monotonically decreasing function of spatial distance. We tested multiple candidate functions with a common set of length-scales used to parameterize them for the crosslinking probability (Supplementary Figure S3) and chose the hyperbolic tangent function (Figure S3C) as it provided the most robust results for the power-law fits of contact

frequency versus genomic distance. Given these assumptions and a set of loci(j)-loci(j') distances, $\mathsf{R}_{j,j'}$, the total probability of crosslinking between loci, $\mathsf{P}_{j,j'}$, is the probability of the union of all possible crosslinking events

$$\mathsf{P}_{j,j'}(\mathsf{R}_{j,j'}) \propto \sum_{k=1}^{Q_{j,j'}} p_{cl}(r_k^{j,j'}). \tag{S40}$$

If this procedure is repeated for a set of $N_{\text{replicates}}$ replicates each containing $N_{\text{timesteps}}$ timesteps, the total crosslinking probabilities can be calculated an ensemble- and/or time-average over these sets of configurational states. When calculating the *in silico* contact maps we implicitly assume that the successful crosslinking would be followed by further processing steps (Crémazy et al., 2018) with 100% efficiency and therefore directly equate the loci(j)-loci(j') crosslinking probabilities, $\mathsf{P}_{j,j'}$, with the loci(j)-loci(j') relative contact frequencies, $\mathsf{F}_{j,j'}$. In a final processing step, all contact maps are then normalized (Cournac et al., 2012) to be doubly-stochastic matrices using the matrix-balancing method of Knight and Ruiz (Knight and Ruiz, 2012), where after normalization rows and columns each sum to one

$$\sum_{j} \mathsf{F}_{j,j'} = 1 \quad \text{and} \quad \sum_{j'} \mathsf{F}_{j,j'} = 1. \tag{S41}$$

To improve visual clarity and contrast, for all contact maps plotted using a log-scale within this work, after all zeros are replaced with the minimum non-zero value, the lower-limit of the colorbar is set to the 10th percentile of contact frequencies observed in the map.

Martini Visualization

Visualization of an entire Syn3A cell in the Martini representation was performed using Visual Molecular Dynamics (VMD) (Humphrey et al., 1996). Working with a structure of nearly 100 million atoms required non-standard VMD practices. Here, we will only discuss the non-standard practices used to generate the render of the Martini representation (Figure 9). We will divide these into three tasks: (1) *Loading a large structure* — After loading the Gromacs structure file (.gro) into VMD, we first exported the structure as a ".js" file, a custom file format that is optimized for VMD and enables rapid loading of coordinates in the future. (2) *Processing atom selections* — The atom selection for each representation in VMD is processed individually. Furthermore, each term within a boolean expansion in the "atom selection" window is evaluated sequentially, which may potentially lead to billions of iterations. Ideally, one should collapse all selections sharing a common visual representation into a single selection and use regular expressions (`resname "A|B|C"` instead of `resname A B C`) within the selection to reduce loop executions. To differentiate atoms of different types combined in the same selection, we then set the representation to color by "resname" and adjust the resname colors in the "Graphics/Colors" menu. If selections involve geometric calculations (ex. membrane cutaway in Figure 9), these selections can be made persistent to avoid recalculation by assigning them to an unused property such as "segname". (3) *Choosing visual representations* — Due to the size of the structure, the OpenGL window will lag as changes are made to the visualization, this is true even for simple tasks such as rotating the structure. Choosing visual representations that do not require additional calculations and reduce the number of polygons drawn in the OpenGL window helps to minimize this behavior. We used the "points" representation for all selections while editing the visualization. Further improvements can be gained by hiding all selections that are not being actively modified. Once all selections have been modified to the user's satisfaction, change each selection from the "points" to the final visual representation before using the "File/Render" function. We chose to use the "TachyonL-OptiX (interactive, GPU-accelerated)" renderer so that we could fine-tune the orientation of the structure for the final render.

Supplementary Tables

Table S1. Software used for simulation, analysis, and visualization.

Program	Description	Web	Reference
`sc_chain_generation`	initial configurations	github.com/brg4/sc_chain_generation	this study
`btree_chromo`	chromosome simulations	github.com/brg4/btree_chromo	this study
`btree_contacts`	contact maps	github.com/brg4/btree_contacts	this study
`LAMMPS`	MD simulation engine	lammps.org	Thompson et al. (2022)
`GROMACS`	MD simulation engine	gromacs.org	Páll et al. (2020)
`Martini`	coarse-grained MD	cgmartini.nl	Souza et al. (2021)
`Polyply`	Martini backmapping	github.com/marrink-lab/polyply_1.0	Grünewald et al. (2022)
`VMD`	MD visualization	ks.uiuc.edu/Research/vmd	Humphrey et al. (1996)

Table S2. Walltimes for a representative selection of simulations included in study, simulation details can be found in their respective sections. All timings were determined by running on 64 threads of a server with two Intel Xeon Gold 6154 CPUs at 3.00 GHz and averaging over five replicate simulations. Replicate simulations were run using scripting capabilities within `btree_chromo`.

Simulation	CPU-time per replicate (hh:mm:ss)
diffusion without bonds (Section 3.1)	214:18:02
diffusion with bonds (Section 3.1)	224:07:15
case i - 0 loops, without topoisomerase (Section 3.2)	486:09:17
case iv - 0 loops, with topoisomerase (Section 3.2)	784:18:08
case vi - 20 loops, with topoisomerase (Section 3.2)	908:59:18

Supplementary Figures

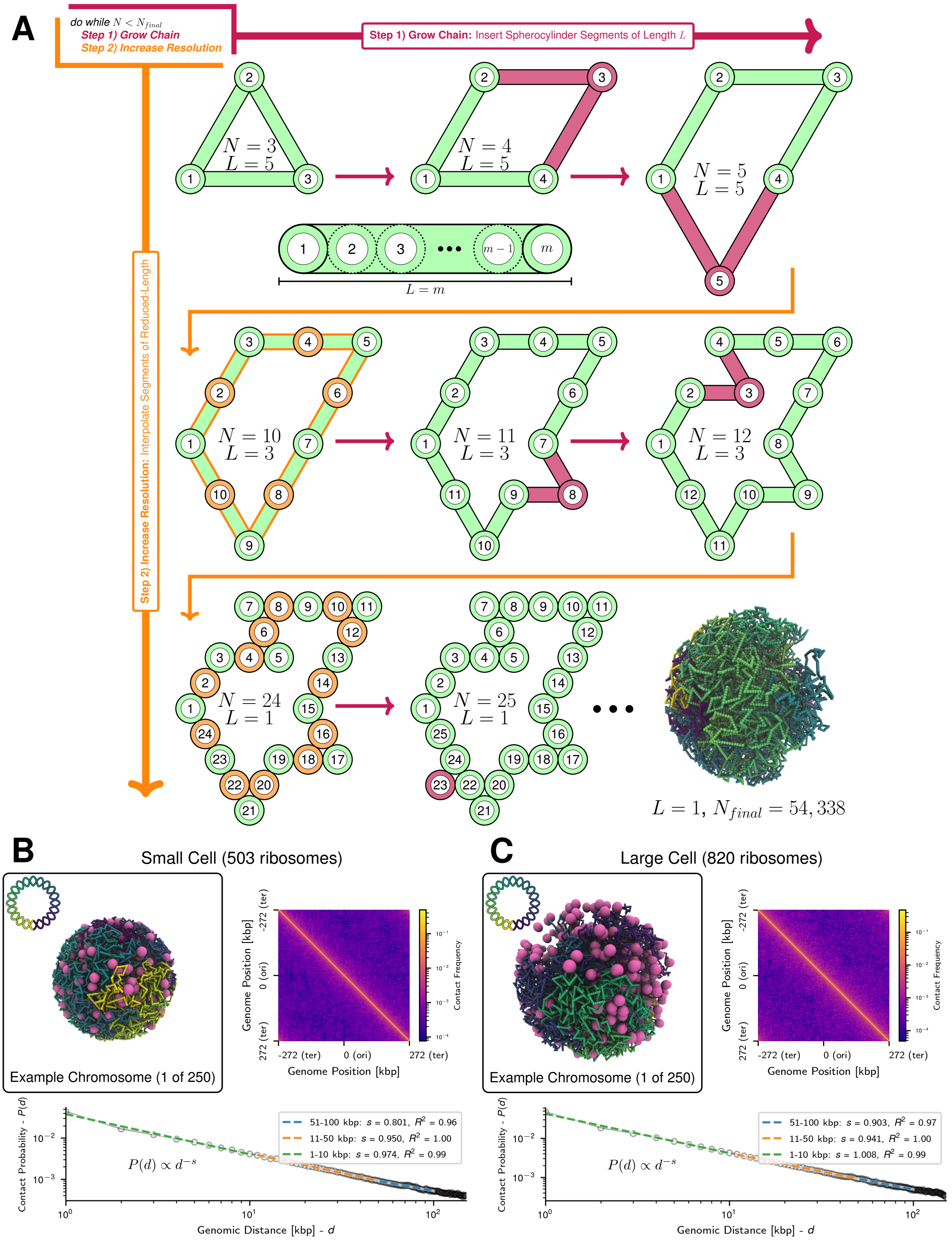

Figure S1. A) Schematic of algorithm using midpoint-displacements to generate initial conditions of chromosomes organized as fractal globules. B) Initial configurations of single unreplicated chromosome in small Syn3A cell and ribosome distribution from (Gilbert et al., 2021). C) Initial configurations of single unreplicated chromosome in large Syn3A cell and ribosome distribution from (Gilbert et al., 2021).

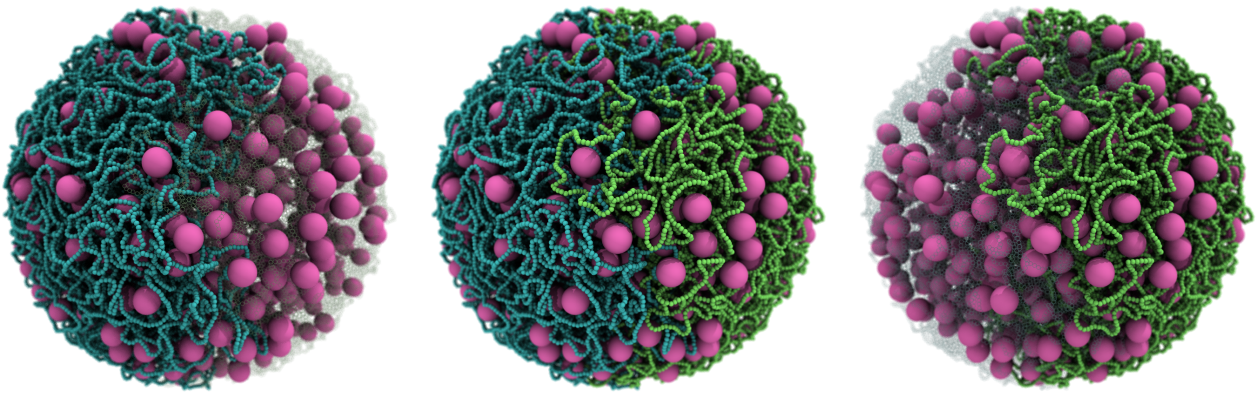

Figure S2. Spherical 250 nm radius cell containing 1000 ribosomes (pink) filled with two 543 kbp chromosomes (blue and green) organized as fractal globules. On the left and right one of the chromosomes are alternatively made transparent to better highlight the well-defined interface between the two chromosomes that nearly bisects the spherical shape and shows a low degree of mixing. The two chromosomes were relaxed from their initial state by minimizing their energy while obeying the energy function.

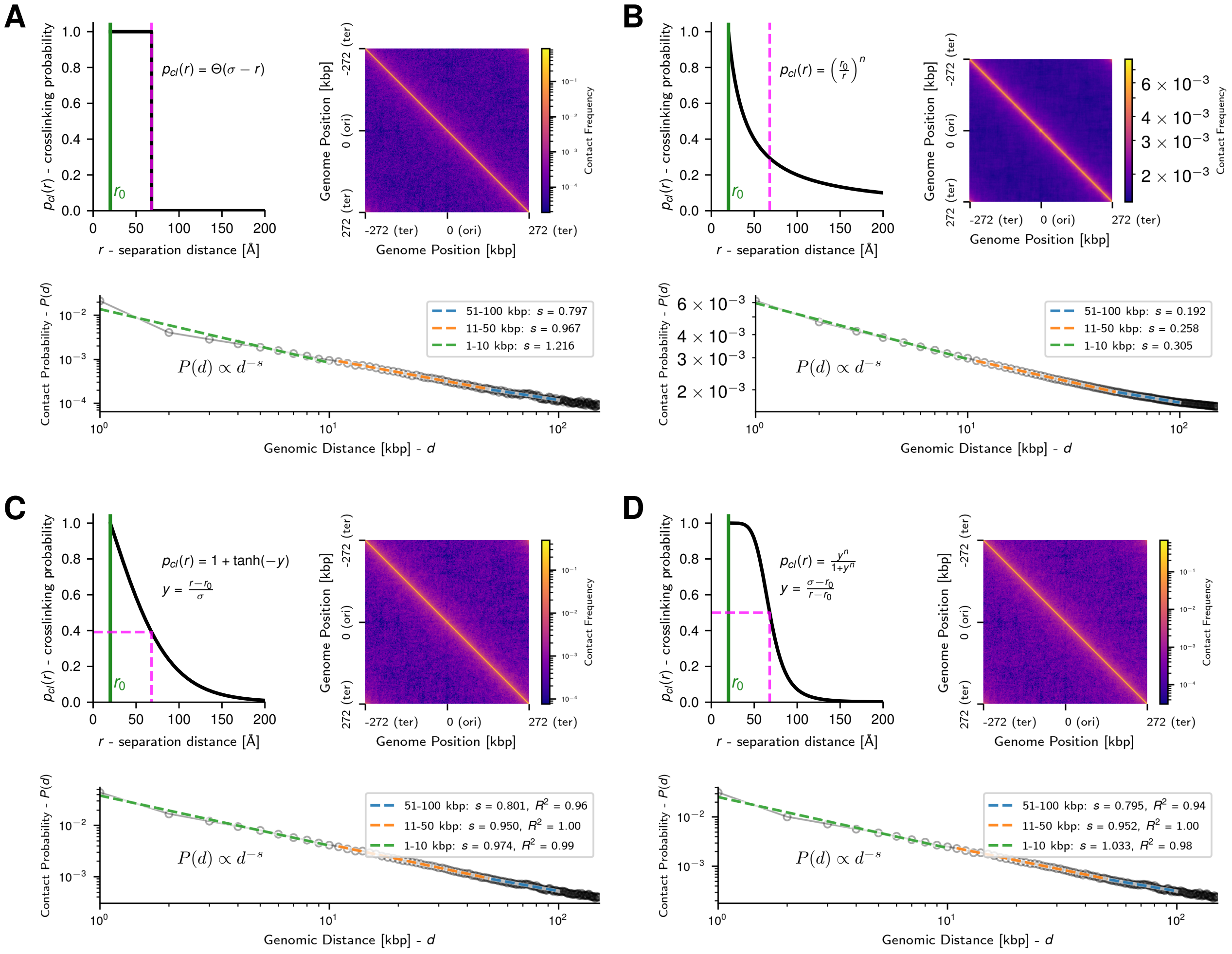

Figure S3. A) Step function crosslinking function with $\sigma = 4r_{\text{DNA}}$. B) Inverse power crosslinking function with $r_0 = r_{\text{DNA}}$ and $n = 1$. C) Hyperbolic tangent crosslinking function with $r_0 = r_{\text{DNA}}$ and $\sigma = 4r_{\text{DNA}}$. D) Hill-type crosslinking function with $r_0 = r_{\text{DNA}}$, $\sigma = 4r_{\text{DNA}}$, and $n = 4$.

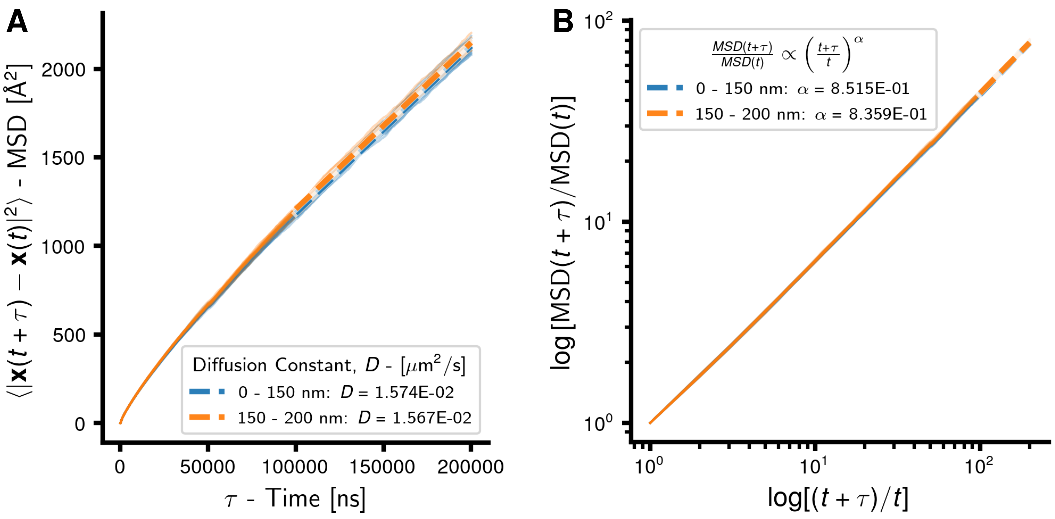

Figure S4. 10 replicates of a system with an unreplicated 54,338 monomer chromosome in 200 nm radius cell with no ribosomes were simulated. The initial conditions were generated in the absence of ribosomes acting as obstacles. A) Brownian diffusion of DNA monomers. B) Anomalous diffusion of DNA monomers.

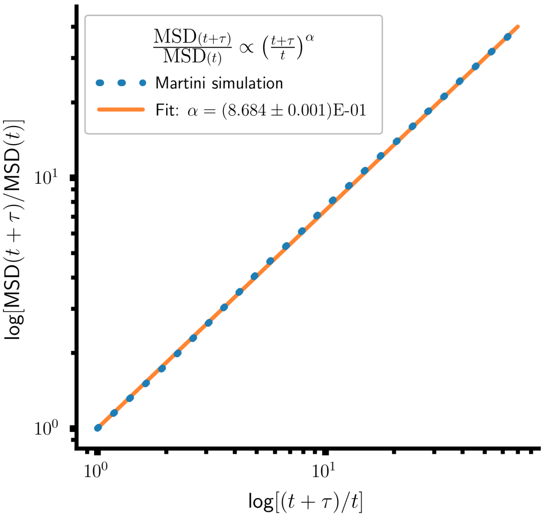

Figure S5. Power-law fit for anomalous diffusion of 10 bp monomer segments' centers of mass in Martini simulation of 50,000 bp toy model (Figure 10A).

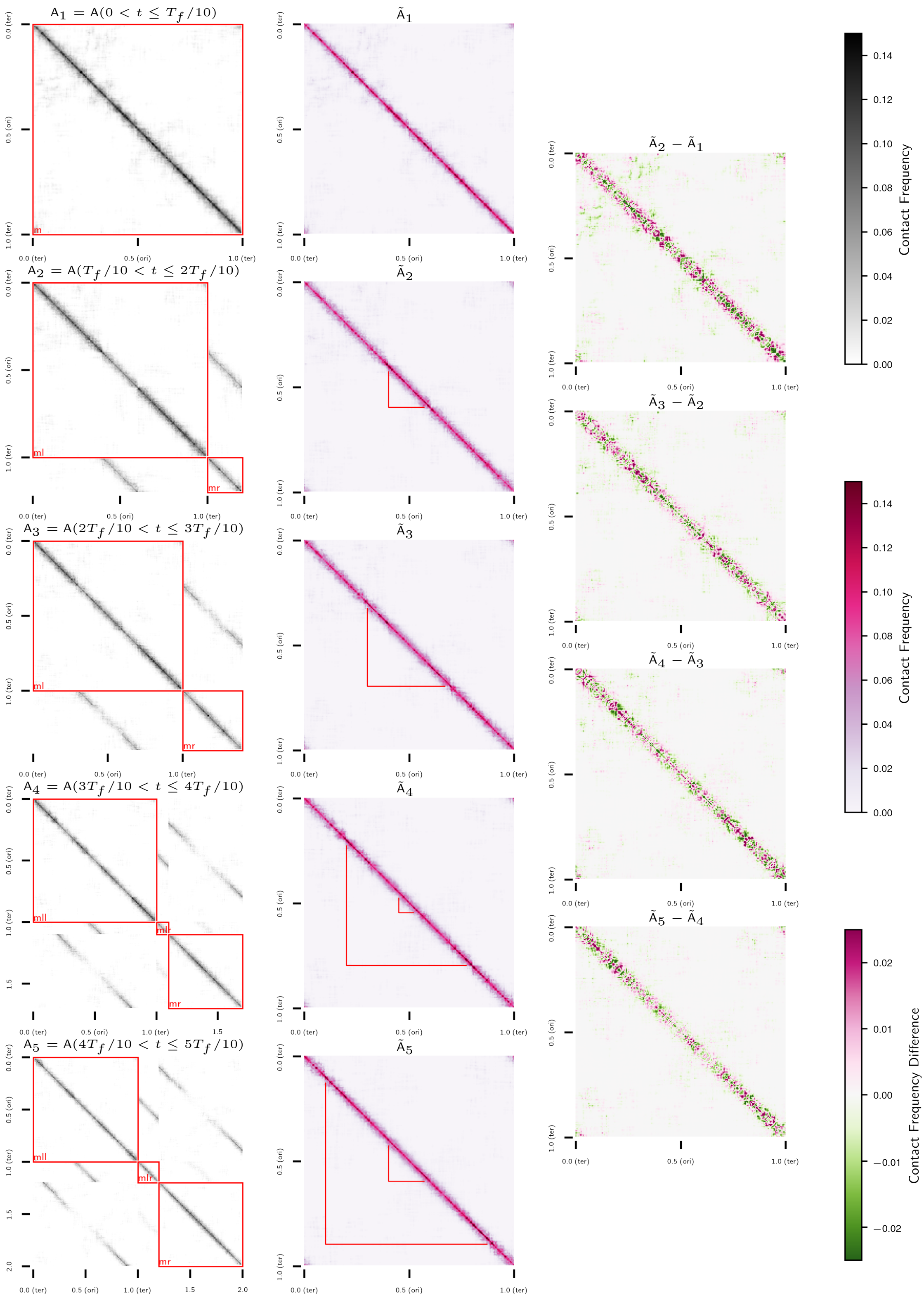

Figure S6. True contact maps (A_i), sequence-equivalent contact maps ($\tilde{A}_i$), and sequence-equivalent map differences ($\tilde{A}_{i+1} - \tilde{A}_i$) of case vi (20 loops, with topoisomerase) for the series of replication states visited by the system (Figure 6A) in the time interval $0 < t \leq 5T_f/10$ (see Supplementary Figure S7 for the other time interval). Red lines in true and sequence-equivalent maps indicate regions containing intra-daughter contacts.

(continued from previous figure)

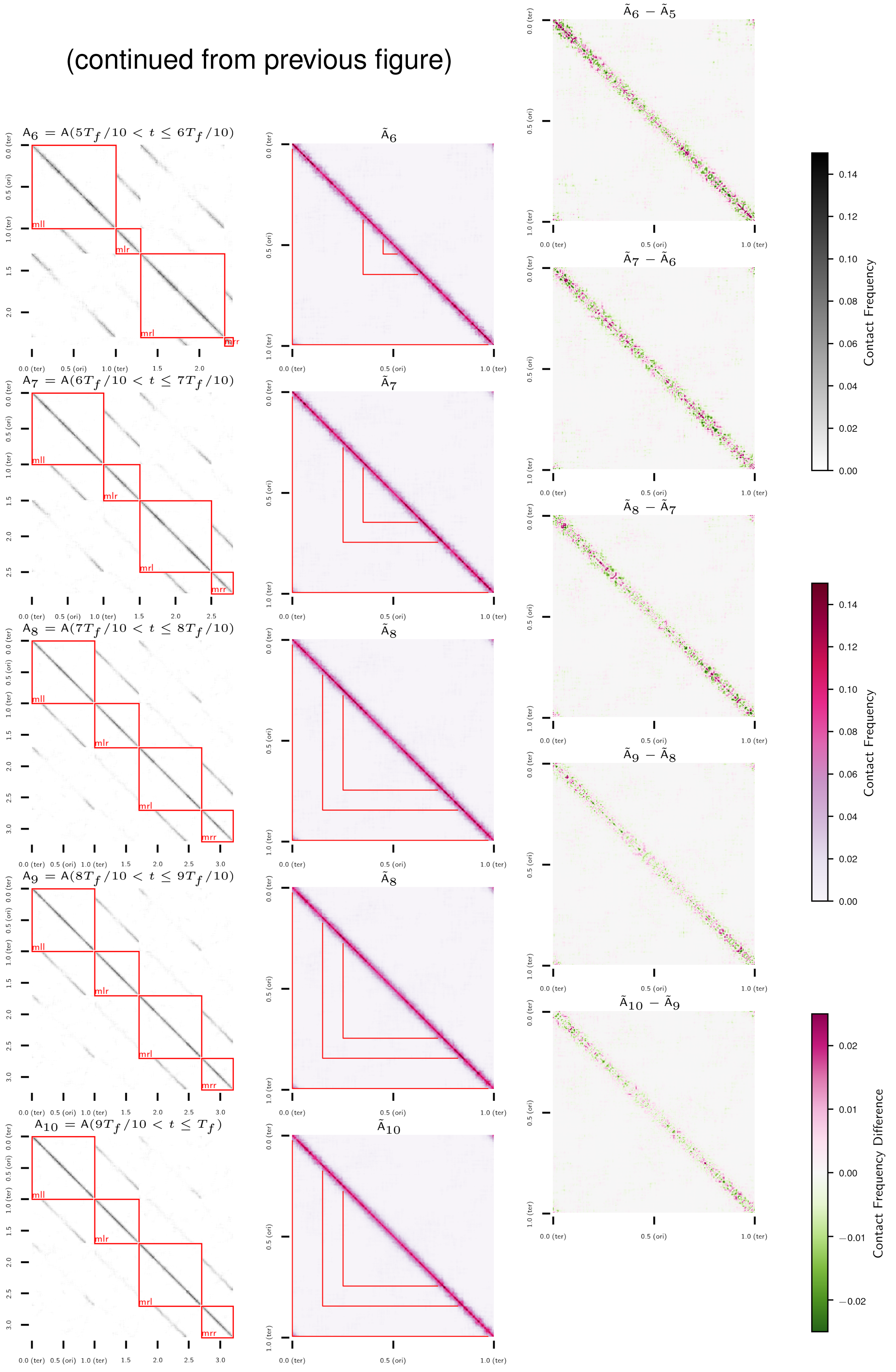

Figure S7. True contact maps (A_i), sequence-equivalent contact maps ($\tilde{\mathrm{A}}_i$), and sequence-equivalent map differences ($\tilde{\mathrm{A}}_{i+1} - \tilde{\mathrm{A}}_i$) of case vi (20 loops, with topoisomerase) for the series of replication states visited by the system (Figure 6A) in the time interval $5T_f/10 < t \leq T_f$ (see Supplementary Figure S6 for the other time interval). Red lines in true and sequence-equivalent maps indicate regions containing intra-daughter contacts.

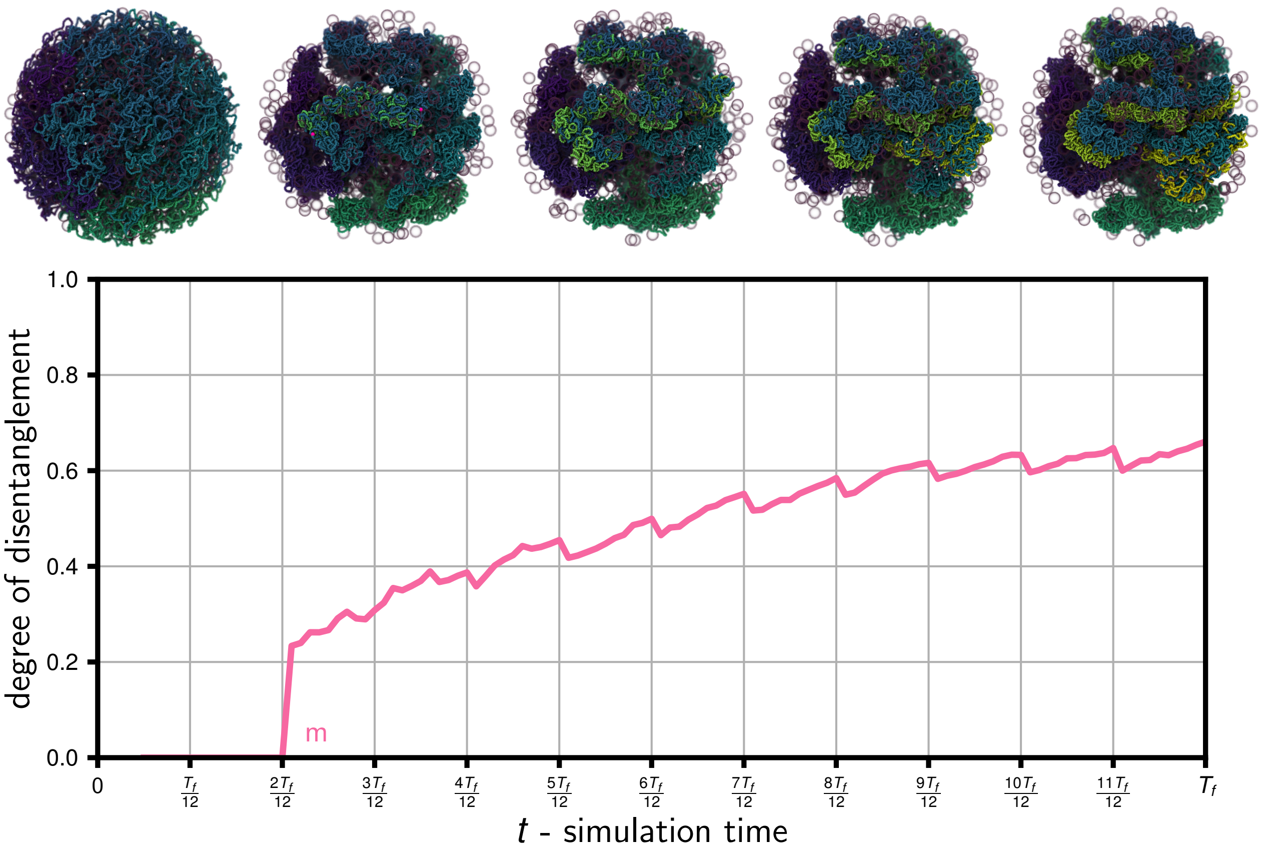

Figure S8. Degree of disentanglement for proof-of-concept simulation of full Syn3A chromosome (54,338 monomers) undergoing simultaneous replication and disentanglement of daughter chromosomes while under the influence of loop-extruding SMC protein complexes and topoisomerases allowing strand-passage (Supplementary Video SV2). The system is simulated for 12.0E+6 total timesteps and replication in 2,000 monomer steps begins after the initial 2.0E+6 timesteps. Representative snapshots at the corresponding times are displayed above the graph.

Supplementary Algorithms

Algorithm 1: Loop updating algorithm

input : $\{\boldsymbol{x}_i(t)\}$ - current coordinates
$N_{\text{loop}}, \{(a_i, h_i, d_i)\}$ - current loop bonds
output : $N_{\text{loop}}, \{(a'_i, h'_i, d'_i)\}$ - updated loop bonds
parameters: L_{min} - minimum loop length
R_g - grab radius
$L_{\text{ext-avg}}$ - average extrusion length
$L_{\text{ext-max}}$ - maximum extrusion length
p_{unbind} - hinge unbinding probability

`// iterate over the current loops`
for $i = 1$ **to** N_{loop} **do**
  `// unbind the hinge with the given probability`
  if $U(0,1) < p_{unbind}$ **then**
    `// get the inter-strand update candidates from the current loop and coordinates`
    $N_{\text{inter}}, \{\lambda_i\}_{\text{inter}} =$ `get_inter_candidates` $(a_i, \{\boldsymbol{x}_i(t)\}, R_g, L_{\text{min}})$
    if $N_{inter} > 0$ **then**
      `// sample a hinge update from inter-candidates with uniform weights`
      $\lambda =$ `sample_inter_candidates` $(N_{\text{inter}}, \{\lambda_i\}_{\text{inter}})$
      `// choose a new extrusion direction at random`
      $\delta = \pm 1$ with equal probability
    else
      `// hinge remains unbound`
      $\lambda = \emptyset$
      $\delta = \emptyset$
    end
  else
    `// get the intra-strand update candidates from the current loop and coordinates`
    $N_{\text{intra}}, \{\lambda_i\}_{\text{intra}} =$ `get_intra_candidates` $(a_i, h_i, d_i, \{\boldsymbol{x}_i(t)\}, R_g, L_{\text{min}}, L_{\text{ext-max}})$
    if $N_{inter} > 0$ **then**
      `// sample a hinge update from intra-candidates from the provided distribution`
      $\lambda =$ `sample_intra_candidates` $(N_{\text{intra}}, \{\lambda_i\}_{\text{intra}}, L_{\text{ext-avg}}, L_{\text{ext-max}})$
      `// maintain the current extrusion direction`
      $\delta = d_i$
    end
  end
  `// update the loop bonds`
  $a'_i = a_i$
  $h'_i = \lambda$
  $d'_i = \delta$
end

Algorithm 2: SMC looping algorithm

input : $\{\boldsymbol{x}_i(t = T_0)\}$ - initial coordinates
output : $\{\boldsymbol{x}_i(t = T_f)\}$ - final coordinates
parameters: M_{loops} - number of loops
T_f - final time
Δt_{loops} - duration of loop simulations before update
T_{topo} - topoisomerase simulation frequency
Δt_{topo} - duration of topoisomerase simulation

// initialize DNA loops
`initialize_loops` (M_{loops})
// initialize simulation time
$t = T_0$
// initialize previous topoisomerase time
$\tau_{\text{topo}} = t$
// main simulation loop
while $t < T_f$ **do**
  // update DNA loops
  `update_loops`
  // determine time increment
  $\Delta t = \min(T_f - t, \Delta t_{\text{loops}})$
  // check for topoisomerase action
  if $(t+\Delta t\ ) \geq (\tau_{\text{topo}} + T_{topo})$ **then**
    // minimize to relax over-stretched bonds from updated loops
    `minimize_topoDNA_harmonic`
    `minimize_topoDNA_FENE`
    // run system under topoisomerase action permitting strand-crossings
    `run_topoDNA_FENE` (Δt_{topo})
    // minimize to relax possible overlaps from incomplete strand-crossings
    `minimize_soft_harmonic`
    `minimize_soft_FENE`
    // advance previous topoisomerase time
    $\tau_{\text{topo}} = t + \Delta t$
  end
  // minimize to relax over-stretched bonds from updated loops
  `minimize_hard_harmonic`
  `minimize_hard_FENE`
  // run the looped system
  `run_hard_FENE` (Δt)
  // advance the simulation time
  $t = t + \Delta t$
end

Supplementary Videos

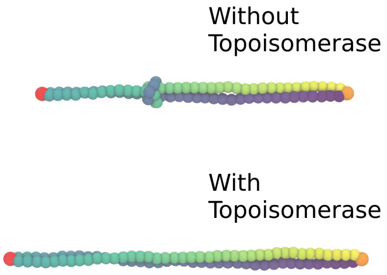

Figure SV1. Video of a circular dsDNA polymer in a trefoil knot being pulled taut with and without the action of topoisomerases. Equal and opposite forces are applied to the *Ori* (red) and *Ter* (orange) for both cases. In the case with topoisomerases, the soft topoisomerase potentials are switched on for a brief interval beginning at the final third of the video (≈10s video time), strand-passages then occur and unknot the polymer.

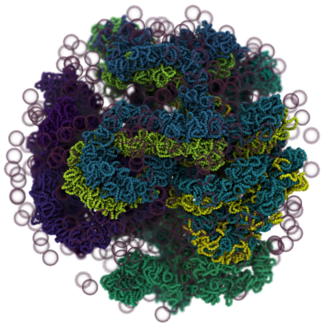

Figure SV2. Video of a full Syn3A chromosome (54,338 monomers) undergoing simultaneous replication and disentanglment of daughter chromosomes while under the influence of loop-extruding SMC protein complexes and topoisomerases allowing strand-passage. The replicating chromosome is colored in accordance with the scheme used throughout the paper (Figure 2A), where the newly replicated right daughter is colored with yellow/green. Forks are magenta, *Ori*s are red, and *Ter*s are orange. The 500 ribosomes in the system are represented as transparent spheres. The video begins with a single unreplicated chromosome that has not previously been subject to looping. The video proceeds to show replication of 20,000 monomers (200 kbp) in 2,000 monomer (20 kbp) steps with segregation occuring simultaneously.

Bibliography

Abbas, A., He, X., Niu, J., Zhou, B., Zhu, G., Ma, T., et al. (2019). Integrating hi-c and FISH data for modeling of the 3d organization of chromosomes. *Nature Communications* 10. doi:10.1038/s41467-019-10005-6

Cournac, A., Marie-Nelly, H., Marbouty, M., Koszul, R., and Mozziconacci, J. (2012). Normalization of a chromosomal contact map. *BMC Genomics* 13. doi:10.1186/1471-2164-13-436

Crémazy, F. G., Rashid, F.-Z. M., Haycocks, J. R., Lamberte, L. E., Grainger, D. C., and Dame, R. T. (2018). Determination of the 3d genome organization of bacteria using hi-c. In *Methods in Molecular Biology* (Springer New York). 3–18. doi:10.1007/978-1-4939-8675-0_1

Eimer, W. and Pecora, R. (1991). Rotational and translational diffusion of short rodlike molecules in solution: Oligonucleotides. *The Journal of Chemical Physics* 94, 2324–2329. doi:10.1063/1.459904

Gilbert, B. R., Thornburg, Z. R., Lam, V., Rashid, F.-Z. M., Glass, J. I., Villa, E., et al. (2021). Generating chromosome geometries in a minimal cell from cryo-electron tomograms and chromosome conformation capture maps. *Frontiers in Molecular Biosciences* 8, 644133. doi:10.3389/fmolb.2021.644133

Grünewald, F., Alessandri, R., Kroon, P. C., Monticelli, L., Souza, P. C. T., and Marrink, S. J. (2022). Polyply; a python suite for facilitating simulations of macromolecules and nanomaterials. *Nature Communications* 13. doi:10.1038/s41467-021-27627-4

Hoffman, E. A., Frey, B. L., Smith, L. M., and Auble, D. T. (2015). Formaldehyde crosslinking: A tool for the study of chromatin complexes. *Journal of Biological Chemistry* 290, 26404–26411. doi:10.1074/jbc.r115.651679

Humphrey, W., Dalke, A., and Schulten, K. (1996). VMD: Visual molecular dynamics. *Journal of Molecular Graphics* 14, 33–38. doi:10.1016/0263-7855(96)00018-5

Knight, P. A. and Ruiz, D. (2012). A fast algorithm for matrix balancing. *IMA Journal of Numerical Analysis* 33, 1029–1047. doi:10.1093/imanum/drs019

Le Treut, G., Képès, F., and Orland, H. (2018). A polymer model for the quantitative reconstruction of chromosome architecture from HiC and GAM data. *Biophysical Journal* 115, 2286–2294. doi:10.1016/j.bpj.2018.10.032

Lieberman-Aiden, E., van Berkum, N. L., Williams, L., Imakaev, M., Ragoczy, T., Telling, A., et al. (2009). Comprehensive mapping of long-range interactions reveals folding principles of the human genome. *Science* 326, 289–293. doi:10.1126/science.1181369

MacKay, K. and Kusalik, A. (2020). Computational methods for predicting 3d genomic organization from high-resolution chromosome conformation capture data. *Briefings in Functional Genomics* 19, 292–308. doi:10.1093/bfgp/elaa004

Meluzzi, D. and Arya, G. (2012). Recovering ensembles of chromatin conformations from contact probabilities. *Nucleic Acids Research* 41, 63–75. doi:10.1093/nar/gks1029

Muñoz-Gil, G., Volpe, G., Garcia-March, M. A., Aghion, E., Argun, A., Hong, C. B., et al. (2021). Objective comparison of methods to decode anomalous diffusion. *Nature Communications* 12. doi:10.1038/s41467-021-26320-w

Oliveira, F. A., Ferreira, R. M. S., Lapas, L. C., and Vainstein, M. H. (2019). Anomalous diffusion: A basic mechanism for the evolution of inhomogeneous systems. *Frontiers in Physics* 7. doi:10.3389/fphy.2019.00018

Páll, S., Zhmurov, A., Bauer, P., Abraham, M., Lundborg, M., Gray, A., et al. (2020). Heterogeneous parallelization and acceleration of molecular dynamics simulations in GROMACS. *The Journal of Chemical Physics* 153, 134110. doi:10.1063/5.0018516

Patrone, P. N. and Rosch, T. W. (2017). Beyond histograms: Efficiently estimating radial distribution functions via spectral monte carlo. *The Journal of Chemical Physics* 146, 094107. doi:10.1063/1.4977516

Press, W. H., Teukolsky, S. A., Vetterling, W. T., and Flannery, B. P. (2007). *Numerical Recipes 3rd Edition: The Art of Scientific Computing* (USA: Cambridge University Press), 3 edn.

Serra, F., Stefano, M. D., Spill, Y. G., Cuartero, Y., Goodstadt, M., Baù, D., et al. (2015). Restraint-based three-dimensional modeling of genomes and genomic domains. *FEBS Letters* 589, 2987–2995. doi:10.1016/j.febslet.2015.05.012

Souza, P. C. T., Alessandri, R., Barnoud, J., Thallmair, S., Faustino, I., Grünewald, F., et al. (2021). Martini 3: a general purpose force field for coarse-grained molecular dynamics. *Nature Methods* 18, 382–388. doi:10.1038/s41592-021-01098-3

Thompson, A. P., Aktulga, H. M., Berger, R., Bolintineanu, D. S., Brown, W. M., Crozier, P. S., et al. (2022). LAMMPS - a flexible simulation tool for particle-based materials modeling at the atomic, meso, and continuum scales. *Computer Physics Communications* 271, 108171. doi:10.1016/j.cpc.2021.108171

Tirado, M. M., Martínez, C. L., and de la Torre, J. G. (1984). Comparison of theories for the translational and rotational diffusion coefficients of rod-like macromolecules. application to short DNA fragments. *The Journal of Chemical Physics* 81, 2047–2052. doi:10.1063/1.447827

Zhang, Z., Li, G., Toh, K.-C., and Sung, W.-K. (2013). 3d chromosome modeling with semi-definite programming and hi-c data. *Journal of Computational Biology* 20, 831–846. doi:10.1089/cmb.2013.0076
